# Sustainable Low-Volume Analysis of Environmental Samples by Semi-Automated Prioritization of Extracts for Natural Product Research (SeaPEPR)

**DOI:** 10.3390/md18120649

**Published:** 2020-12-17

**Authors:** Riyanti, Michael Marner, Christoph Hartwig, Maria A. Patras, Stevy I. M. Wodi, Frets J. Rieuwpassa, Frans G. Ijong, Walter Balansa, Till F. Schäberle

**Affiliations:** 1 Institute for Insect Biotechnology, Justus-Liebig-University of Giessen, 35392 Giessen, Germany; riyanti@bio.uni-giessen.de; 2 Faculty of Fisheries and Marine Science, Jenderal Soedirman University, Purwokerto 53122, Indonesia; 3 Fraunhofer Institute for Molecular Biology and Applied Ecology (IME), Branch for Bioresources, 35392 Giessen, Germany; Michael.Marner@ime.fraunhofer.de (M.M.); Christoph.Hartwig@ime.fraunhofer.de (C.H.); Maria.Patras@ime.fraunhofer.de (M.A.P.); 4 Department of Fisheries and Marine Science, Politeknik Negeri Nusa Utara, Tahuna Sangihe Islands, North Sulawesi 95812, Indonesia; wodiimelda@gmail.com (S.I.M.W.); frets.jr@gmail.com (F.J.R.); ijongfrans@yahoo.com (F.G.I.); 5 Faculty of Fisheries and Marine Science, Sam Ratulangi University, Manado 95115, Indonesia; 6 German Center for Infection Research (DZIF), Partner Site Giessen-Marburg-Langen, 35392 Giessen, Germany

**Keywords:** natural products, dereplication, antibiotics, marine sponges, plant pathogen

## Abstract

The discovery of novel natural products (NPs) that will serve as lead structures has to be an ongoing effort to fill the respective development pipelines. However, identification of NPs, which possess a potential for application in e.g., the pharma or agro sector, must be as cost effective and fast as possible. Furthermore, the amount of sample available for initial testing is usually very limited, not least because of the fact that the impact on the environment, i.e., the sampled biosystem, should be kept minimal. Here, our pipeline SeaPEPR is described, in which a primary bioactivity screening of crude extracts is combined with the analysis of their metabolic fingerprint. This enabled prioritization of samples for subsequent microfractionation and dereplication of the active compounds early in the workflow. As a case study, 76 marine sponge-derived extracts were screened against a microbial screening panel. Thereunder, human pathogenic bacteria (*Escherichia coli* ATCC35218 and *Staphylococcus aureus* ATCC33592) and yeast (*Candida albicans* FH2173), as well as the phytopathogenic fungus *Septoria tritici* MUCL45407. Overall, nine extracts revealed activity against at least one test organism. Metabolic fingerprinting enabled assigning four active extracts into one metabolic group; therefore, one representative was selected for subsequent microfractionation. Dereplication of the active fractions showed a new dibrominated aplysinopsin and a hypothetical chromazonarol stereoisomer derivative. Furthermore, inhibitory activity against the common plant pest *Septoria tritici* was discovered for NPs of marine origin.

## 1. Introduction

Natural products (NPs) are the oldest form of medicine utilized by humans. Technologies and methods improved and NPs remained one of the most important source for the development of medicinal drugs. Today, NPs and their derivatives make up a significant percentage of approved drugs worldwide. Especially in the antibiotics sector, almost all lead structures were identified from bio-resources (~75% either unaltered or semi-synthetically modified; 1981–2014) [1,2]. Although being a traditional source for antimicrobial compounds, the pool of NP-derived structural novelty is not exhausted as exemplified by the discovery of teixobactin [3] and darobactin [4].

Besides clinical application, specialized natural products are drivers of socio-economic stability by finding application in food preservation, livestock and aquaculture treatment, as well as crop protection [5]. In all fields, humans benefit from the evolutionary shaped intrinsic antimicrobial activity of NPs. In the 1940s, the “Waksman antibiotic discovery platform” was the first systematical approach to identify antimicrobial NPs and led to the isolation of the first aminoglycosides [6].

However, high rediscovery rates make classical discovery campaigns unattractive and pose an unreasonable financial risk for the private sector. This might partly be circumvented by implementing chemo-informatics in systematic, routine processes. Data mining processes such as automatic annotation of bucket matrices [7] or MS/MS networks [8] against public databases help to identify signals and interest, even in gigantic datasets.

Besides discovery of novelty, repurposing of already known structures to different fields of application seems an encouraging approach, but high expense or no commercial availability of many natural products reduce feasibility substantially. In this context, SeaPEPR represents a methodology allowing a preliminary determination of specific bioactivity of single compounds within crude environmental extracts. Application allows us to evaluate the bio-economical value of large sets of low volume sample on the metabolite level in a standardized manner and finally facilitates decision making on downstream processes such as isolation of unknown metabolites or repurposing studies.

Here, we chose a promising and likewise challenging bio-resource as a case study to present our approach of crude environmental extract analysis. Sponges, as sessile filter feeders without physical defense, are believed to depend on chemical defense or deterrence mechanisms mediated NPs, biosynthesized either by themselves or by associated microorganisms. Due to ethical reasons, straightforward isolation and characterization of compounds by harvesting sponges from nature should be discouraged. Limited availability of material usually prohibits extensive information retrieval from a given environmental sample. Frequently, scaffolds initially discovered in environmental samples are subject to delicate chemical synthesis without a clear product application. MS/MS coupled microfractionation of environmental extracts facilitates semi-automatic dereplication and allows attributing bioactivity observed in crude extract primary screens to single compounds without the necessity of cost and time-consuming isolation or synthesis.

## 2. Results

### 2.1. Sample Collection and Extract Generation

**Figure 1:**
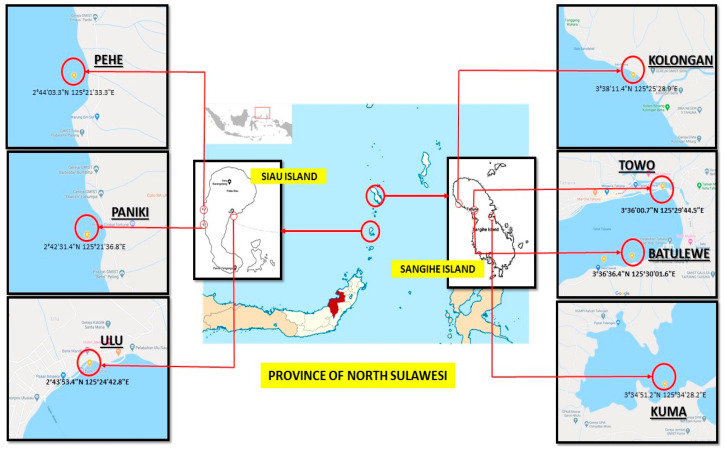
Sampling sides of sponge specimen. Samples were retrieved by SCUBA in a depth of 4–20 m below the surface.

Sponges are a well-known bioresource for bioactive molecules and can be regarded as a complex environmental sample, since the holobiont (consisting of the sponge and its associated microbes) is extracted as a whole. Furthermore, the taxonomic classification of sponges, which is based on both, genetic barcoding and morphology, is time consuming and challenging. In this project, 76 sponge samples from seven different dive sites at the coastal area of Sangihe and Siau Island (Pacific Ocean, Indonesia) were obtained (Figure 1). At each diving site, around 11–15 sponge samples were collected, except sponge sample T_5, which was the only one obtained from the site named Towo. Hence, it can be expected that the sample set represents a survey of the biodiversity around the islands. How this translates into chemical diversity was investigated in the following. As starting material, 5 mg of dried sponge, which is approximately equivalent to the size of a thumbnail, was used. From all samples, crude extracts were prepared. The extraction yield (based on dry mass) using methanol was between 12 and 86% (Figure S1).

**Figure 2:**
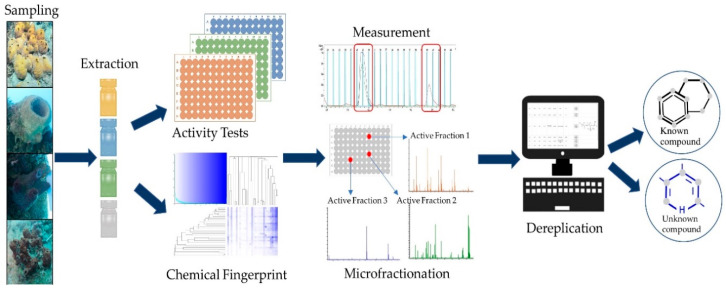
Schematic overview of the SeaPEPR pipeline. In a first step, crude environmental extracts are subject of bioactivity assessment. At the same time, the chemical diversity of the entire set of samples is determined by cosine similarity calculation (“chemical fingerprints”). Prioritized samples are microfractionated to identify the causative agent responsible for the initially observed bioactivity. If desired, dereplicated compounds of interest can be selected for isolation.

The generated extracts represent the material further analyzed for NP discovery. The general workflow of SeaPEPR is depicted in Figure 2.

### 2.2. Bioactivity Assessment—Microbroth Dilution Assays

In order to determine the antimicrobial potency, the generated extracts were screened against a diverse panel of pathogenic microorganisms including *Escherichia coli* ATCC35218, *Staphylococcus aureus* ATCC33592, *Pseudomonas aeruginosa* ATCC27853, as well as *Candida albicans* FH2173 and the phytopathogenic fungus *Septoria tritici* MUCL45407. In total, seven of the 76 tested sponge extracts exhibited growth inhibitory effects of at least 85% across 3 dilution steps against one or more test strains and were thereby considered bioactive: Essentially, samples KOL_8, KOL_16, KOL_18, and ULU_13 were active against *S. aureus*, *C. albicans*, and *S. tritici*, PEHE_5 against *S. aureus* and *S. tritici*, and extracts PANIKI_4 and ULU_16 showed activity only against *S. tritici*. In addition, two extracts (ULU_11 and ULU_17) showed weak activity against *C. albicans* by inhibiting the cell viability of the test strain only in the highest concentrations. No growth inhibition of the selected Gram-negative test strains was observed.

### 2.3. Prioritization—Metabolic Fingerprinting

**Figure 3:**
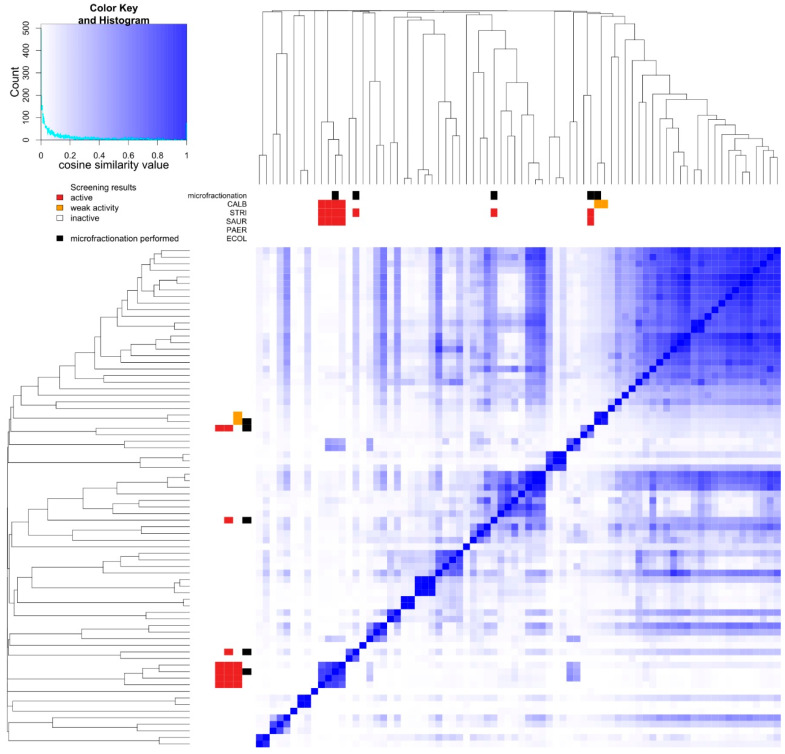
Cosine similarity heatmap of all 76 extracts. Blue color indicates a high degree of similarity among compared extracts (see color key histogram). Flags in sidebar mark selected samples for microfractionation (black) and screening results (red = active, orange = weak activity, white = inactive) of the respective extract against the indicator strains (CALB = *C. albicans*, STRI = *S. tritici*, SAUR = *S. aureus*, PAER = *P. aeruginosa*, ECOL = *E. coli*).

**Figure 4:**
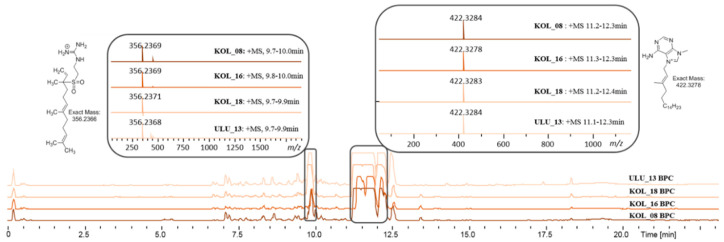
Base peak chromatogram (BPC) of the extracts KOL_08, KOL_16, KOL_18, and ULU_13 obtained from different *Agelas nakamurai* organisms. Most intense peaks within the similar BPCs correspond to the agelasines groups (box, top right) and agelasidine A (box, top left).

Results from metabolic fingerprinting and bioactivity screening were combined to allow prioritization of samples and are summarized in Figure 3. The detailed grouping results with pairwise similarities are presented in Table S1. While a total of 45 distinct metabolic groups was generated, extracts sharing the same activity pattern (KOL_8, KOL_16, KOL_18, and ULU_13) were assigned to the same group (Figure 3), strongly suggesting a similar metabolite composition of the extracts (see also Figure 4). Similarly, extracts ULU_11 and ULU_17 formed one group, while extracts PEHE_5, PANIKI_4, and ULU_16 appeared to consist of unique metabolite mixtures. From each metabolic group containing bioactive extracts, one representative was selected for microfractionation. Samples selected for microfractionation are marked.

### 2.4. Dereplication of Bioactive Compounds—Microfractionation

#### 2.4.1. KOL_18 (TSRR0002_D-07) *Agelas nakamurai*

**Figure 5:**
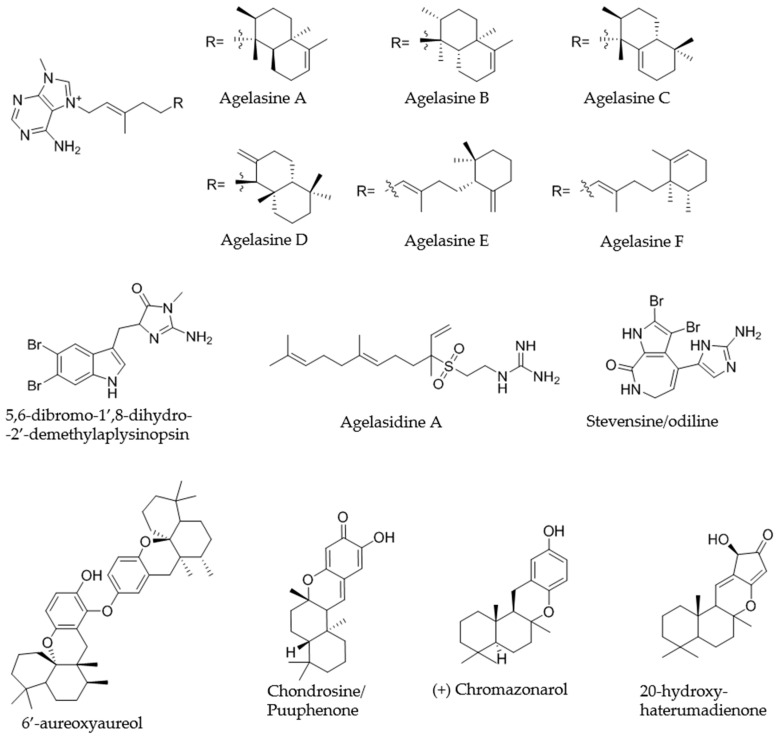
Chemical structures of the dereplicated compounds responsible for the activity of the microfractionated samples.

Extract KOL_18 was selected as representative of the group of the four extracts exhibiting an identical bioactivity pattern and similar metabolite composition (Figure 4). Based on the primary activity of the crude extract against *S. aureus*, the corresponding crude extract (1 mg/mL in MeOH) was fractionated in 1 and 2 µL injection volume replicates and rescreened against the same indicator strain. The active fractions were reproduced in both dilutions. The two activity zones, namely fractions 80–81 and 83–84 (Figure S2), could be assigned to partly co-eluting isomeric compounds with an *m/z* 422.3283 [M]^+^, corresponding to the molecular formula [C_26_H_40_N_5_]^+^. The compounds showed an UV absorption at 220 and 272 nm. Based on the MS/MS fragmentation pattern, the compounds could be assigned as members of the agelasine A-F family (Figure 5) [9]; since MS/MS fragmentation does not allow for distinction between the different isomeric structures of the diterpene unit. As the name indicates, agelasines are known metabolites of the sponge *Agelas nakamurai*.

The same extract was fractionated against *S. tritici* (injection volume 2 and 5 µL). Both of the replicates showed activity, corresponding to the above-described agelasines. The 5 µL injection volume replicate showed an additional activity zone, namely fraction 69 (Figure S3), which could be assigned to a compound of *m/z* 356.2370 [M + H]^+^, corresponding to the molecular formula C_18_H_33_N_3_O_2_S_1_. The compound shows UV absorption at 220 nm. Based on the MS/MS fragmentation pattern (Figure S3d), the compound was dereplicated as agelasidine A [10] (Figure 5), also a known metabolite of *A. nakamurai* [11]. The extract was also fractionated against *C. albicans* (2 mg/mL solution, injection volume 2 and 5 µL). Both injection replicates showed activity in the fractions corresponding to the above described agelasines (A-F) and agelasidine A (Figure S4).

Molecular networking analysis revealed the presence of several derivatives (minor compounds) including oxo-agelasines (A-F) of *m/z* 436.3073 [M]^+^ with a molecular formula of C_26_H_38_N_5_O_1_, hydroxy-agelasines of *m/z* 438.3231 [M]^+^ with a molecular formula of C_26_H_40_N_5_O_1_, and dihydro-hydroxy-agelasines of *m/z* 440.3389 [M]^+^ with a molecular formula of C_26_H_42_N_5_O_1_, each present in the extract as a complex mixture of isomers (Figures S5 and S6).

#### 2.4.2. PEHE_5 (TSRR0002_F-08) *Haliclona* sp.

Based on the results of the primary screening against *S. aureus*, the corresponding crude extract (1 mg/mL in MeOH) was fractionated in 2 and 5 µL injection volume replicates and rescreened against the same indicator strain. Only the 5 µL replicate showed active fractions, namely fractions 47–48 and fraction 108 (Figures S7 and S8).

Activity of fractions 47-48 was assigned to a compound of *m/z* 398.9449 [M + H]^+^ showing the specific isotope pattern of a dibrominated compound (Figure S7b) corresponding to a molecular formula of C_13_H_13_Br_2_N_4_O_1_. The compound shows UV absorption maxima at 220 and 292 nm. The fragmentation pattern is indicative of a dibrominated triptamine framework as structural subunit. A substructure search on SciFinder retrieved no hits corresponding to the assigned molecular formula, however, one candidate, namely 5,6-dibromo-2′-demethylaplysinopsin (C_13_H_11_Br_2_N_4_O_1_ - one additional degree of unsaturation compared to the compound in the extract), was found to fit the fragmentation pattern observed for the compound in the extract. Therefore, the active compound was putatively assigned the structure of 5,6-dibromo-1′,8-dihydro-2′-demethylaplysinopsin [12] (Figure 5), which is in agreement with the molecular formula, fragmentation pattern, and observed UV spectrum. Aplysinopsins are a family of indole alkaloids isolated from sponges [13], scleractinian corals [14,15] and sea anemones [16]. The tentative 5,6-dibromo-1′,8-dihydro-2′-demethylaplysinopsin is, to our knowledge, not reported in literature.

Activity of fraction 108 was assigned to a mixture of co-eluting compounds of *m/z* 627.4414 [M + H]^+^, *m/z* 315.2321 [M + H]^+^, *m/z* 329.2115 [M + H]^+^ (Figure S8b–d; proposed structures in Figure 5), corresponding to the molecular formulae C_42_H_58_O_4_, C_21_H_30_O_2_, and C_21_H_28_O_3_, respectively. The compounds showed UV absorption maxima at 223 and 299 nm. The MS/MS fragmentation pattern of all three compounds showed a base peak ion of *m/z* 191.1798, corresponding to the molecular formula of C_14_H_23_^+^, which was assigned to the retro Diels–Alder fragmentation product ion of sesquiterpene hydroquinone frameworks. Based on the MS/MS data for C_21_H_30_O_2_, no distinction between the two literature known isomeric sponge metabolites, aureol and chromazonarol [17,18], could be made. The same holds true for C_42_H_58_O_4_ (putatively 6′-aureoxyaureol or 6′-aureoxychromazonarol). The bissesquiterpene 6′-aureoxyaureol was reported together with several dibrominated aplysinopsin derivatives in *Smenospongia* sp., whereas the hypothetical chromazonarol stereoisomer was not described [19]. Finally, literature query of C_21_H_28_O_3_, produced a range of hits corresponding to algal metabolites, while only one compound, chondrosine (a.k.a. puupehenone) [20], was previously reported from sponges. The structures of 6′-aureoxyaureol, chondrosine, and chromazonarol (Figure 5) were chosen as representative examples for each of the ions of *m/z* 627.4414, *m/z* 329.2115, and *m/z* 315.2321, respectively.

The same extract was fractionated against *S. tritici* (injection volume 2 and 5 µL), however, no active fractions could be observed.

#### 2.4.3. ULU_16 (TSRR0002_H-07) *Neopetrosia* sp.

To investigate the observed antifungal activity of ULU_16, the extract was fractionated in 2 and 5 µL injection volume replicates and rescreened against *S. tritici*. The activity zone was reproduced in the two replicates and could be assigned to a compound of *m/z* 385.9249 [M + H]^+^ showing the specific isotope pattern of a dibrominated compound (Figure S9) with the molecular formula C_11_H_9_Br_2_N_5_O_1_. The compound showed UV absorption maxima at 220 and 340 nm. Based on the MS/MS fragmentation pattern, the compound was dereplicated as stevensine, also known as odiline (Figure 5), a metabolite reported in various sponge species [21].

#### 2.4.4. PANIKI_4 (TSRR0002_D-12) *Halichondria* sp.

The microfractionated extract PANIKI_4 (injection volume 1 and 2 µL, 1 mg/mL) was rescreened against *S. tritici*. Only the 2 µL injection volume replicate produced one active zone (Figure S10), which could be assigned to a compound of *m/z* 317.2112 [M + H]^+^ corresponding to the molecular formula of C_20_H_28_O_3._ The fragmentation pattern of the compound does not allow for clear assignment of substructural frameworks. The UV absorption maximum was detected at 220 nm. A database search of the molecular formula retrieved a sesquiterpene compound, namely 20-hydroxyhaterumadienone (Figure 5), as plausible candidate for tentative structure assignment. 20-hydroxyhaterumadienone is a cytotoxic compound reported from *Dysidea* sp. [22].

#### 2.4.5. ULU_11 (TSRR0002_H-03)

The extract ULU_11 was selected as a representative of extracts exhibiting weak *C. albicans* activity and highly similar metabolite composition (together with ULU_17, Figure 3). The crude extract was fractionated in 2 and 5 µL injection volume replicates and rescreened against *C. albicans*. However, no active fractions could be identified.

## 3. Discussion

In this study, we used a set of 76 sponge samples to present our Semi-automated Prioritization of Extracts for natural Product Research (SeaPEPR) pipeline. Primary bioactivity assessment led to the identification of nine sponge extracts exhibiting bioactivity against at least one of the selected indicator strains. Simultaneously, unsupervised chemical diversity visualization by cosine similarity heat map construction facilitated the overall data interpretation and prioritization of extracts for downstream processes.

**Figure 6:**
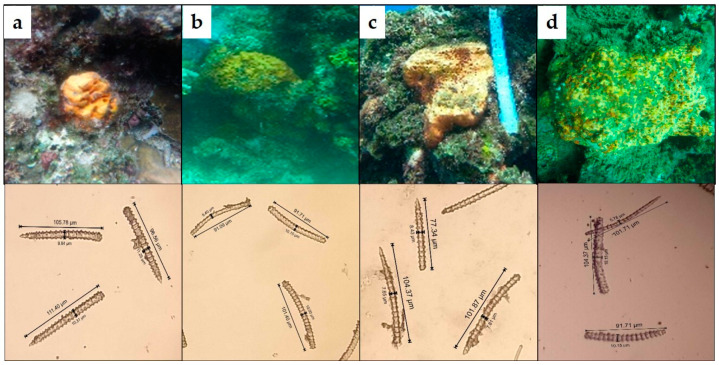
Underwater pictures and isolated spicules of the *Agelas nakamurai* cf specimens (**a**) Sample KOL_8, (**b**) Sample KOL_16, (**c**) Sample KOL_18, and (**d**) Sample ULU_13. It can be seen that the specimens are thick encrusting orange sponges and the type of spicule is megascleres acanthostyle for all four samples. This suggested the assignment as *Agelas nakamurai* cf.

During prioritization, four bioactive extracts (KOL_08, KOL_16, KOL_18, and ULU_13) were grouped together, indicating highly similar metabolite composition. In fact, orthogonal data obtained from sponge identification by morphological features, such as spicule identification (Figure 6, Table S2), indicated taxonomic uniformity of the organisms (i.e., all specimens were identified as *A. nakamurai* based on spicule morphology). In this case, taxonomic uniformity translated into chemical uniformity. Consequently, agelasines and agelasidines (dereplicated in the active fractions of the representative extract KOL_18) were found in all members of this metabolic group (Figure 3).

On the other hand, PEHE_5 and PANIKI_4 were initially taxonomically classified as members of the genus *Haliclona*. In contrast, the metabolite fingerprinting of these two sponges clearly indicated distinctiveness of organisms. A focused investigation on morphological level finally revealed that PANIKI_4 belongs to the genus *Halichondria*. Six other sponges were morphologically identified as *Haliclona* sp. However, only two pairs of high metabolic similarity could be observed in the heat map, indicating different *Haliclona* species. Within this genus, speciation seems to be tightly linked to chemical diversification, as *Haliclona* extracts did not cluster, but were distributed throughout the heat map. It is known that besides species affiliation of the holobiont, the chemical profile could also be shaped by the associated microbial communities [23], the habitat [24], as well as stress associated to predation and wounding [25].

Both observations, chemical uniformity within a species (*A. nakamurai*) and interspecies metabolic diversity (*Haliclona* sp.) can be explained by the well-accepted assumption that taxonomic, thus genetic, diversity is often expressed by chemical diversity. Broad chemical diversity is generally desired in natural product discovery campaigns and thereby careful selection of the source material is crucial. In this context, the prioritization of extracts based on the similarity of their chemical composition helps to maximize metabolite diversity in downstream processes. Especially for samples for which reliable species identification in the field (e.g., sponges) is challenging, chemotyping (e.g., cosine similarity heatmaps) as interface between primary screenings and follow up experiments seems useful to decrease workload. Besides, it has to be kept in mind that even different intra-species samples have the potential for the detection of new and even novel compounds, since analysis of the same species could result in different metabolomes due to the dynamic environmental factors [26]. Independent from the sample set, it demands a straightforward downstream pipeline to mine the vast amount of data. While other microfractionation platforms are suitable to acquire detailed information about extracts obtained from precisely selected samples such as different medicinal plants [27,28], one benefit of the herein presented pipeline is the potential to characterize extracts (and not necessarily the source organism) in detail without processing replicates and yet account for most drivers of metabolic diversity. After prioritization, extract components (ions) are directly linked to the observed bioactivity. Other elegant strategies (e.g., bioactive molecular networking [29]) establish this connection by calculation of the Pearson correlation between the relative abundance of ions across chromatographic fractions (usually 18–20) and the observed bioactivity. Our alternative dereplication approach aims to screen fractions containing only a very limited number of, if not single, ions or ions all belonging to the same molecular feature against the indicators strain (Figures S3, S7–S10). Because fraction collection in assay plates is coupled to MS/MS, a direct, experimental connected between candidate molecule and bioactivity can be established. By using this workflow, five out of initially 76 extracts were prioritized based on bioactivity and unique metabolic fingerprint, before the causative metabolites were determined by microfractionation.

Bioactivity of extracts obtained from *A. nakamurai* could be assigned to agelasines and agelasidine A. Synthetic access to the agelasines was already established [30] and broad compound profiling was carried out: Reported bioactivities include Na,K-ATPase inhibition [9], cyto- and ichthyotoxicitiy, antiprotozoal [31], and antifouling activity, as well as growth inhibition of *M. tuberculosis*, Gram-positive and negative pathogenic bacteria [32], as well as yeast (reviewed by Gordaliza) [33]. Likewise, agelasidines were observed to exhibit activity against *S. aureus* and *C. albicans* [11]. Broad screening of aplysinopsins demonstrated a modulating activity against the glycine-gated chloride channel receptor [13], antineoplastic, antiplasmodial, anti-bacterial, as well as anti-fungal activities. The latter included growth inhibition of *Penicillium atrovenetum* and *Trichophyton mentagrophytes* (reviewed by Bialonska and Zjawiony) [12]. Besides aplysidine A, a mix of several cytotoxic [17,18] sesquiterpene hydroquinones was dereplicated in the extract PEHE_5 obtained from *Haliclona* sp. The bioactivity of *Neopetrosia* sp. extract ULU_16 was attributed to stevensine (odiline). Reported activity of stevensine comprises fish deterrence [34] and weak antimicrobial growth inhibition (e.g., *Deleya marina*, a common fouling bacterium) [35]. The compound 20-hydroxyhaterumadienone (here dereplicated from PANIKI_4 a putative *Halichondria* sp.) is known to possess cytotoxic effects [22,36], and exhibit weak interaction with human lipoxygenase (5-hLO) [37].

While these results indicate a generally robust transfer of primarily observed growth inhibitory effects to microfractionated assays, two extracts did not show bioactivity in any fraction. These findings emphasize a general challenge in bioactivity driven NP research (in contrast to cheminformatics inclined discovery projects [38]): Microbial crude extracts are composed of a mixture of various substances at dramatically different concentrations and potencies. It is important to realize that almost each substance (or a combination of several metabolites) becomes unspecifically toxic at high concentrations, hence producing a positive assay read out. As discrimination between specific and unspecific effects might come at the price of insensitivity, we chose a trade off in favor of false positive instead of false negative results. Consequently, initially moderately active crude extracts (e.g., ULU_11 against *C. albicans*) might not produce positive microfractionation read outs. Given suitable chromatography parameters, members of compound families are separated and tested individually at lower overall concentration. In the case of PEHE_5, the microfractionated extract was unsuccessfully rescreened against *S. tritici.* Potentially, the sum of compounds present in the extract (di-brominated aplysinopsins; aureol/chromazonal) possessed additive, however unspecific, growth inhibition of the test strain, while individual compounds did not show the effect. Although the reduction of unspecific effects caused by high concentration of compounds seems to be an advantage, separation and individual testing of metabolites might also prohibit identification of synergistic effects.

Another limitation of rapid MS/MS-based annotation approaches, including the herein presented methodology, is the reduced identification confidence of target molecules (as defined by the Metabolite Annotation Task Group of the Metabolomics Society) [39,40] compared to full structure and stereochemistry assignment studies. In that sense, no distinction between the isomeric sponge metabolites aureol and chromazonarol or between putatively 6′-aureoxyaureol and 6′-aureoxychromazonarol could be made. Besides these challenges, SeaPEPR has proven its value as prioritization strategy allowing data-based decision making on follow-up projects early in the discovery process. This study gave insight into the metabolites of four morphologically seemingly different specimens of *A. nakamurai*, preventing an otherwise very daunting task of molecular structure elucidation.

If a compound exhibits the desired properties such as structural novelty, repurposing potential, or just the isolation of more material for further in detail investigation of observed bioactivities, the metabolite should undergo further analysis, including confirmation of the 3-dimensional structure and extensive activity profiling. For repurposing studies of small molecules, the required amount (~ 1 mg) to carry out experiments required for hit characterization might be generated by straightforward chemical synthesis as shown for the agelasines [30]. While an unknown and likewise bioactive metabolite is scientifically most intriguing, it initially requires more sample material; hence, detailed metadata should be recorded in the field (Table S2) to allow resupply. Collection of specimens with the same chemotype might be challenging, but not per se, as observed by the robust metabolic fingerprint of *A. nakamurai* across sampling sites (>60 km distance between Kolongan and Ulu sampling sites). Before isolation from animal tissue is conducted, metabolite access via fermentation of the cultivable microbiome should be investigated. If this route is obstructed, authorities should decide case by case whether a targeted isolation campaign from animal tissue, towards new and urgently needed antibiotic or agrochemical lead structures, is ethically justifiable. Selection of promising projects might be facilitated by data obtained from prioritization processes, such as SeaPEPR.

Finally, yet importantly, to the best of our knowledge, no bioactivity against the common plant pest *S. tritici* was reported for any of the herein dereplicated sponge compounds. The ascomycete *S. tritici*, which is the causative agent of blotch disease on wheat, is responsible for serious losses in cereal yields and quality in Western European countries. In 2014, an estimated $1.3 billion worth of fungicides was used to control *Septoria*-induced crop rust [41]. Resistance development, strict EU regulations, and increased public awareness against the use of petrochemicals drive the continuous demand for new agents with potency against *S. tritici*. The herein presented data indicate that marine-derived natural products pose potential solutions for current challenges in plant pest control.

## 4. Materials and Methods

### 4.1. Sponge Collection

Sponges were collected from Paniki (2°42′31.4′′ N, 125°21′36.8′′ E), Pehe (2°44′03.3′′ N, 125°21′33.3′′ E), and Ulu (2°43′53.4′′ N, 125°24′42.8′′ E) of Siau Islands Regency and from Batulewehe (3°36′00.7′′ N, 125°29′44.5′′ E), Kolongan (3°38′11.4′′ N, 125°25′28.9′′ E), and Kuma (3°34′51.2′′ N, 125°34′28.2′′ E) of Sangihe Islands Regency North Sulawesi Indonesia at a depth between ~4 and ~20 m during May 2019. After morphological description and underwater documentation by photograph (GoPro Hero 4.0, except for specimens from Kolongan which were taken by GroPro Hero 7.0), each specimen was cut and kept individually in a plastic bag. Samples were transferred to the laboratory in Politeknik Negeri Nusa Utara Tahuna Indonesia where the specimens were stored at −16 °C until used. From each specimen, a small portion (1 cm^3^) was taken for taxonomic identification using slightly modified bleach digestion method [42,43]. All specimens were individually sliced into small pieces, dried in the oven at 45 °C for 3 days and blended to give either powder or mash of sponges. This drying step was performed, since the infrastructure available at the islands is limited and the material prepared in this way was then ready to be sent by normal post. Sponges of 2 to 5 g were packed individually in a small plastic bag separately secured in 76 sample bottles and sent to Justus-Liebig-University Giessen, Germany on October 2019.

### 4.2. Sample Extraction

From the sponge samples, a portion of 5 mg dry weight was solved in 500 µL of methanol. The sample was cut in small pieces and subsequently macerated in a shaker (140 rpm, 30 °C overnight). In a next step, the debris was pelleted by centrifugation in a table top centrifuge at full speed for 5 min. The supernatant was taken and the remaining material (pellet) was extracted one more time with 500 µL methanol. The supernatants were combined and evaporated under a flow of N_2_, before storage at −20 °C. The dry weight of the extract was determined and extraction efficiency was calculated. The crude extract was dissolved in dimethyl sulfoxide (DMSO, final concentration 1 mg/mL) for the antimicrobial assays and in methanol (1 mg/mL) for LC-HRMS measurement.

### 4.3. Antimicrobial Bioassays

Antimicrobial activity of the crude sponge extracts was determined by micro broth dilution assays in 384 well microtiter plates (Greiner, Kremsmünster, Austria). A Cybi Liquid handling system (Analytic Jena, Jena, Germany) was used to distribute 0.5, 0.25, and 0.125 µL (in duplicate, corresponding to 10.0, 5.0, and 2.5 µg/mL extract concentration) of each extract to the assay plates. A dilution series of gentamycin (64–0.002 µg/mL, Sigma Aldrich, St. Louis, MS, USA) was added to the antibacterial assays as positive control, while wells containing only medium or only bacterial suspension were used as sterile and growth control respectively. Pre-cultures of *E. coli* ATCC35218*, S. aureus* ATCC33592, and *P. aeruginosa* ATCC27853 were incubated (overnight, 37 °C, 180 rpm) in cation adjusted Mueller Hinton II medium (Becton Dickinson, Sparks, NV, USA) before the cell density was adjusted to 2 × 10^4^ cells/mL and 50 µL bacterial suspension was added to each well (except the sterile control) using a multi-well dispenser (Multidrop; Thermo Labsystems, Waltham, MA, USA). After incubation (18 h, 37 °C, 180 rpm, 80% rH), cell growth was assessed by turbidity measurement with a microplate spectrophotometer at 600 nm (LUMIstar^®^ Omega BMG Labtech, Ortenberg, Germany).

The pre culture of *C. albicans* FH2173 was incubated for two days at 27 °C. Cell density was diluted to 1 × 10^5^ cells/mL in Mueller Hinton II medium before the assay plates were incubated for 48 h at 37 °C, 180 rpm, and 80% rH. For *S. tritici* MUCL45407, a previously prepared spore solution was used to adjust the assay inoculum to 1 × 10^5^ spores/mL in YM medium (yeast extract 4 g × L^−1^, malt extract 4 g × L^−1^, sucrose 4 g × L^−1^). *Septoria* assay plates were incubated for 72 h at 24 °C, 180 rpm, and 80% rH. Nystatin (Sigma Aldrich) was used as positive control for both, yeast and mold assays. Cell viability was evaluated via ATP quantification (BacTiter-Glo™, Promega, Madison, WI, USA) according to the manufacturer’s instructions.

### 4.4. UPLC-HRMS/MS and Microfractionation

UHPLC-HR-MS analysis was performed on a 1290 UHPLC system (Agilent, Santa Clara, CA, USA) equipped with DAD, ELSD, and maXis II™ (Bruker, Billerica, MA, USA) ESI-qTOF-UHRMS with the following gradient: 0 min: 95% A; 0.30 min: 95% A; 18.00 min: 4.75% A; 18.10 min: 0% A; 22.50 min: 0% A; 22.60 min: 95% A; 25.00 min: 95% A (A: H2O, 0.1% formic acid (FA); B: Acetonitrile, 0.1% FA; Flow: 600 µL/min). Column oven temperature: 45°C. Column: Acquity UPLC BEH C18 1.7 µm (2.1x100 mm) with Acquity UPLC BEH C18 1.7 µm VanGuard Pre-Column (2.1 × 5 mm).

For microfractionation, the flow path was changed, so that 90% of the flow was collected with a custom made fraction collector (Zinsser–Analytik, Eschborn, Germany) while the rest was analyzed in MS/MS mode in maXis II™. Collision induced fragmentation was performed at 28.0–35.05 eV using argon at 10^−2^ mbar.

Depending on the potency observed in the primary screening, microfractionation assay plates were prepared by injecting 1 and 2 µL or 2 and 5 µL of extract. A total of 159 fractions were generated per extract and collected on one 384 well plate (fraction length is 7 s, starting immediately after injection) (Figure S11). Plates were dried in vacuo using a HT12-II centrifugal concentrator (Genevac, Ipswitch, Suffolk, GB) at 35 °C before screening. Microfractionation assay volume of *S. aureus* and *C. albicans* was 20 µL, while volume of *S. tritici* assays was 50 µL.

### 4.5. Metabolic Fingerprinting

MS Data processing was performed with DataAnalysis 4.4 (Bruker, Billerica, MA, USA) using recalibration with sodium formate (Sigma Aldrich), RecalculateLinespectra (threshold 10,000), and FindMolecularFeatures (0.5–25 min, S/N = 0). Bucketing was performed using ProfileAnalysis 2.3 (Bruker, Billerica, MA, USA) (30–1080 s, *m/z* 100–1600, Advanced Bucketing with Δ12 s and Δ5 ppm, no transformation, Bucketing basis = H^+^). The bucket table was subsequently used as input for analysis via R.R (version 3.6.0) [44] with libraries readr [45], coop [46], gplots [47], data.table [48], parallelDist [49], and devtools [50] were used. For heatmap-generation with several sidebars, a variation of heatmap.2 by Griffith [51] was used. The script used in this publication is deposited on GitHub [repository https://github.com/christoph-hartwig-ime-br/cosine-V3; https://dx.doi.org/10.5281/zenodo.4320539]. For sample comparison, the cosine similarities (dot product of vectors) between samples were calculated. Samples were sorted according to clustering results and pairwise similarities were used to determine metabolic groups. If the pairwise similarity between subsequent clustered samples is 0.7 or higher, they were assigned to one metabolic group.

### 4.6. Dereplication

MS and MS/MS Data analysis was performed with DataAnalysis 4.4 (Bruker, Billerica, MA, USA). Molecular formula assignment was done manually for all compounds present in the active fractions, allowing a mass accuracy tolerance of ± 2 ppm. Annotation of the MS/MS spectra was performed manually for all the compounds present in active fractions, whenever no hits against our pure compound library were observed. Molecular formula searches were performed on AntiBase 2017 [52], Dictionary of Natural Products [53] and SciFinder^®^ [54].

### 4.7. Molecular Networking

The UHPLC-QTOF-MS/MS data of the prioritized extracts were visualized and subsequently analyzed using molecular networking. Established parameters [8,55] were used for the experiment. MSConvert (ProteoWizard package32) was used to convert the raw data (*.d files) into plain text (*.mgf) files, wherein all detected fragment ions are expressed as a list of mass/intensity value pairs sorted according to their parent ions (peak picking: vendor MS level = 1-2; threshold type = absolute intensity, value = 1000, orientation = most-intense). The networking algorithm itself, thus the calculation of cosine similarity values between parent ion vectors, was computed offline, using an in house server [38].

## 5. Conclusions

In summary, a combination of (i) phenotypic activity screening assays and (ii) metabolic fingerprinting allowed a fast prioritization and dereplication of samples with the desired bioactivity for further processing. Applying our SeaPEPR pipeline, we were able to dereplicate the active component(s) of crude extracts responsible for the antimicrobial activities observed in primary screens. Thereby, a new dibrominated aplysinopsin and a hypothetical chromazonarol stereoisomer derivative were dereplicated. Furthermore, inhibitory activity against the common plant pest *S. tritici* was discovered for natural products of marine origin. The pipeline represents a valuable tool for further bioprospecting projects, since only low sample volumes are needed that in turn renders extensive collection of limited bioresources for screening purposes (e.g., slow-growing macroorganisms like sponges) obsolete.

## Supplementary Materials

The following are available online at https://www.mdpi.com/1660-3397/18/12/649/s1, Pairwise similarities of clustered samples and resulting metabolic grouping; Morphological description and underwater documentation; Microfractionation bioassays and dereplication results.
