# Supplementary material for: Sustainable Low-Volume Analysis of Environmental Samples by Semi-Automated Prioritization of Extracts for Natural Product Research (SeaPEPR)
Source: Mar Drugs. 2020 Dec 17;18(12):649. doi: 10.3390/md18120649 (PMC7765863; doi:10.3390/md18120649)
Supplement: Supplementary file 1 [file marinedrugs-18-00649-s001.pdf]

marine drugs

MDPI

Supplementary Materials

Sustainable Low-Volume Analysis of Environmental Samples by Semi-Automated Prioritization of Extracts for Natural Product Research (SeaPEPR)

Riyanti [1,2,3,†], Michael Marner [3,†], Christoph Hartwig [3], Maria A. Patras [3], Stevy I. M. Wodi [4], Frets J. Rieuwpassa [4], Frans G. Ijong [4,5], Walter Balansa [4,*] and Till F. Schäberle [1,3,6,*]

[1] Institute for Insect Biotechnology, Justus-Liebig-University of Giessen, 35392 Giessen, Germany; riyanti@bio.uni-giessen.de
[2] Faculty of Fisheries and Marine Science, Jenderal Soedirman University, Purwokerto 53122, Indonesia;
[3] Fraunhofer Institute for Molecular Biology and Applied Ecology (IME), Branch for Bioresources, 35392 Giessen, Germany; Michael.Marner@ime.fraunhofer.de (M.M.); Christoph.Hartwig@ime.fraunhofer.de (C.H.); Maria.Patras@ime.fraunhofer.de (M.P.)
[4] Department of Fisheries and Marine Science, Politeknik Negeri Nusa Utara, Tahuna Sangihe Islands, North Sulawesi 95812, Indonesia; wodiimelda@gmail.com (S.I.M.W.); frets.jr@gmail.com (F.J.R.); ijongfrans@yahoo.com (F.G.I.)
[5] Faculty of Fisheries and Marine Science, Sam Ratulangi University, Manado 95115, Indonesia
[6] German Center for Infection Research (DZIF), Partner Site Giessen-Marburg-Langen, Giessen, Germany
* Correspondence: walterbalansa@polnustar.ac.id (W.B.); till.f.schaeberle@agrar.uni-giessen.de (T.F.S.); Tel.: +49-641-99-37140 (T.F.S.)
† These authors contributed equally.

Contents

Supplementary Tables

Table S1. Pairwise similarities of clustered samples and resulting metabolic grouping

Table S2. Morphological description of sponges KOL_08, KOL_16, KOL_18 and ULU_13 and underwater documentation

Supplementary Figures

Figure S1. Sponges investigated in this study and yield of extraction

Figure S2. Microfractionation: Bioassay and dereplication results plate KOL_18 against *Staphylococcus aureus*

Figure S3. Microfractionation: Bioassay and dereplication results plate KOL_18 against *Septoria tritici*

Figure S4. Microfractionation: Bioassay and dereplication results plate KOL_18 against *Candida albicans*

Figure S5. Molecular networking of the analyzed sponge sample set

Figure S6. Enlarged cluster of agelasine derivatives and MS/MS fragmentation analysis

Figure S7. Microfractionation: Bioassay and dereplication results plate PEHE_5 against *S. aureus* fractions 47-48

Figure S8. Microfractionation: Bioassay and dereplication results plate PEHE_5 against *S. aureus* fraction 108

Figure S9. Microfractionation: Bioassay and dereplication results plate ULU_16 against *S. triciti*

Figure S10. Microfractionation: Bioassay and dereplication results plate PANIKI_4 against *S. triciti*

Figure S11. Microfractionation: general μ-fractionation plate layout

Table S1. Pairwise similarities of clustered samples and resulting metabolic grouping

Sample	Pairwise cosine	Metabolic group (0.7)	Sample ID
X:\2019-12-05_Schaeberle_A1p_10k_SN0\TSRR0002_F-04_A1p_P2-F-7_01_53159.d	0	1	T_5
X:\2019-12-05_Schaeberle_A1p_10k_SN0\TSRR0002_G-07_A1p_P2-B-2_01_53173.d	0.845572753	1	ULU_1
X:\2019-12-05_Schaeberle_A1p_10k_SN0\TSRR0002_E-03_A1p_P2-E-5_01_53144.d	0.817301893	1	PANIKI_5
X:\2019-12-05_Schaeberle_A1p_10k_SN0\TSRR0002_E-11_A1p_P2-F-4_01_53156.d	0.804278199	1	PANIKI_18
X:\2019-12-05_Schaeberle_A1p_10k_SN0\TSRR0002_H-06_A1p_P2-C-2_01_53182.d	0.846885255	1	ULU_15
X:\2019-12-05_Schaeberle_A1p_10k_SN0\TSRR0002_D-11_A1p_P2-E-3_01_53142.d	0.930428595	1	PANIKI_3
X:\2019-12-05_Schaeberle_A1p_10k_SN0\TSRR0002_G-03_A1p_P2-A-7_01_53169.d	0.833846083	1	PEHE_10
X:\2019-12-05_Schaeberle_A1p_10k_SN0\TSRR0002_D-03_A1p_P2-D-4_01_53133.d	0.803911482	1	KOL_13
X:\2019-12-05_Schaeberle_A1p_10k_SN0\TSRR0002_F-11_A1p_P2-A-5_01_53167.d	0.735445223	1	PEHE_8
X:\2019-12-05_Schaeberle_A1p_10k_SN0\TSRR0002_A-03_A1p_P2-A-1_01_53101.d	0.659071771	2	BL_1
X:\2019-12-05_Schaeberle_A1p_10k_SN0\TSRR0002_E-07_A1p_P2-E-9_01_53150.d	0.637927943	3	PANIKI_10
X:\2019-12-05_Schaeberle_A1p_10k_SN0\TSRR0002_E-04_A1p_P2-E-6_01_53145.d	0.690402335	4	PANIKI_6
X:\2019-12-05_Schaeberle_A1p_10k_SN0\TSRR0002_F-12_A1p_P2-A-6_01_53168.d	0.9172631	4	PEHE_9
X:\2019-12-05_Schaeberle_A1p_10k_SN0\TSRR0002_D-09_A1p_P2-E-1_01_53140.d	0.678517726	5	PANIKI_1
X:\2019-12-05_Schaeberle_A1p_10k_SN0\TSRR0002_F-03_A1p_P2-F-6_01_53158.d	0.895923176	5	PANIKI_20
X:\2019-12-05_Schaeberle_A1p_10k_SN0\TSRR0002_E-12_A1p_P2-F-5_01_53157.d	0.834843678	5	PANIKI_19
X:\2019-12-05_Schaeberle_A1p_10k_SN0\TSRR0002_G-05_A1p_P2-A-9_01_53171.d	0.677195099	6	PEHE_14
X:\2019-12-05_Schaeberle_A1p_10k_SN0\TSRR0002_E-05_A1p_P2-E-7_01_53148.d	0.622802274	7	PANIKI_8
X:\2019-12-05_Schaeberle_A1p_10k_SN0\TSRR0002_G-09_A1p_P2-B-4_01_53175.d	0.495296877	8	ULU_4
X:\2019-12-05_Schaeberle_A1p_10k_SN0\TSRR0002_G-12_A1p_P2-B-7_01_53178.d	0.441072394	9	ULU_9
X:\2019-12-05_Schaeberle_A1p_10k_SN0\TSRR0002_F-06_A1p_P2-F-9_01_53161.d	0.528518763	10	PEHE_2
X:\2019-12-05_Schaeberle_A1p_10k_SN0\TSRR0002_G-08_A1p_P2-B-3_01_53174.d	0.287752804	11	ULU_2
X:\2019-12-05_Schaeberle_A1p_10k_SN0\TSRR0002_C-03_A1p_P2-C-3_01_53122.d	0.16868076	12	KM_11
X:\2019-12-05_Schaeberle_A1p_10k_SN0\TSRR0002_G-04_A1p_P2-A-8_01_53170.d	0.147234059	13	PEHE_12
X:\2019-12-05_Schaeberle_A1p_10k_SN0\TSRR0002_G-06_A1p_P2-B-1_01_53172.d	0.266894781	14	PEHE_15
X:\2019-12-05_Schaeberle_A1p_10k_SN0\TSRR0002_H-08_A1p_P2-C-4_01_53184.d	0.047730056	15	ULU_17
X:\2019-12-05_Schaeberle_A1p_10k_SN0\TSRR0002_H-03_A1p_P2-B-8_01_53179.d	0.878330315	15	ULU_11
X:\2019-12-05_Schaeberle_A1p_10k_SN0\TSRR0002_F-08_A1p_P2-A-2_01_53164.d	0.020499965	16	PEHE_5
X:\2019-12-05_Schaeberle_A1p_10k_SN0\TSRR0002_B-09_A1p_P2-B-8_01_53117.d	0.487292669	17	KM_6
X:\2019-12-05_Schaeberle_A1p_10k_SN0\TSRR0002_C-12_A1p_P2-D-3_01_53132.d	0.039901317	18	KOL_11
X:\2019-12-05_Schaeberle_A1p_10k_SN0\TSRR0002_B-10_A1p_P2-B-9_01_53118.d	0.641326389	19	KM_8
X:\2019-12-05_Schaeberle_A1p_10k_SN0\TSRR0002_F-07_A1p_P2-A-1_01_53163.d	0.01406283	20	PEHE_4
X:\2019-12-05_Schaeberle_A1p_10k_SN0\TSRR0002_D-10_A1p_P2-E-2_01_53141.d	0.962507941	20	PANIKI_2
X:\2019-12-05_Schaeberle_A1p_10k_SN0\TSRR0002_C-08_A1p_P2-C-8_01_53127.d	0.747722085	20	KOL_5
X:\2019-12-05_Schaeberle_A1p_10k_SN0\TSRR0002_G-10_A1p_P2-B-5_01_53176.d	0.241174937	21	ULU_6
X:\2019-12-05_Schaeberle_A1p_10k_SN0\TSRR0002_G-11_A1p_P2-B-6_01_53177.d	0.973911588	21	ULU_7
X:\2019-12-05_Schaeberle_A1p_10k_SN0\TSRR0002_F-09_A1p_P2-A-3_01_53165.d	0.877792718	21	PEHE_6
X:\2019-12-05_Schaeberle_A1p_10k_SN0\TSRR0002_C-04_A1p_P2-C-4_01_53123.d	0.600764918	22	KM_13
X:\2019-12-05_Schaeberle_A1p_10k_SN0\TSRR0002_D-05_A1p_P2-D-6_01_53135.d	0.427158951	23	KOL_15
X:\2019-12-05_Schaeberle_A1p_10k_SN0\TSRR0002_A-09_A1p_P2-A-7_01_53107.d	0.722789893	23	BL_9

X:\2019-12-05_Schaeberle_A1p_10k_SN0\TSRR0002_B-05_A1p_P2-B-4_01_53113.d	0.719881971	23	KM_2
X:\2019-12-05_Schaeberle_A1p_10k_SN0\TSRR0002_H-07_A1p_P2-C-3_01_53183.d	0.30835053	24	ULU_16
X:\2019-12-05_Schaeberle_A1p_10k_SN0\TSRR0002_B-04_A1p_P2-B-3_01_53112.d	0.282645433	25	BL_15
X:\2019-12-05_Schaeberle_A1p_10k_SN0\TSRR0002_C-11_A1p_P2-D-2_01_53131.d	0.733995291	25	KOL_9
X:\2019-12-05_Schaeberle_A1p_10k_SN0\TSRR0002_A-10_A1p_P2-A-8_01_53108.d	0.535876053	26	BL_10
X:\2019-12-05_Schaeberle_A1p_10k_SN0\TSRR0002_E-10_A1p_P2-F-3_01_53155.d	0.023532767	27	PANIKI_14
X:\2019-12-05_Schaeberle_A1p_10k_SN0\TSRR0002_C-09_A1p_P2-C-9_01_53128.d	0.076270264	28	KOL_6
X:\2019-12-05_Schaeberle_A1p_10k_SN0\TSRR0002_A-12_A1p_P2-B-1_01_53110.d	0.721063739	28	BL_13
X:\2019-12-05_Schaeberle_A1p_10k_SN0\TSRR0002_C-07_A1p_P2-C-7_01_53126.d	0.620897476	29	KOL_3
X:\2019-12-05_Schaeberle_A1p_10k_SN0\TSRR0002_H-05_A1p_P2-C-1_01_53181.d	0.524667283	30	ULU_14
X:\2019-12-05_Schaeberle_A1p_10k_SN0\TSRR0002_E-08_A1p_P2-F-1_01_53153.d	0.348892857	31	PANIKI_11
X:\2019-12-05_Schaeberle_A1p_10k_SN0\TSRR0002_B-12_A1p_P2-C-2_01_53121.d	0.945814485	31	KM_10
X:\2019-12-05_Schaeberle_A1p_10k_SN0\TSRR0002_B-06_A1p_P2-B-5_01_53114.d	0.927905842	31	KM_3
X:\2019-12-05_Schaeberle_A1p_10k_SN0\TSRR0002_B-11_A1p_P2-C-1_01_53120.d	0.074030661	32	KM_9
X:\2019-12-05_Schaeberle_A1p_10k_SN0\TSRR0002_C-05_A1p_P2-C-5_01_53124.d	0.966532397	32	KM_15
X:\2019-12-05_Schaeberle_A1p_10k_SN0\TSRR0002_F-10_A1p_P2-A-4_01_53166.d	0.036839827	33	PEHE_7
X:\2019-12-05_Schaeberle_A1p_10k_SN0\TSRR0002_D-04_A1p_P2-D-5_01_53134.d	0.760303799	33	KOL_14
X:\2019-12-05_Schaeberle_A1p_10k_SN0\TSRR0002_E-06_A1p_P2-E-8_01_53149.d	0.027309927	34	PANIKI_9
X:\2019-12-05_Schaeberle_A1p_10k_SN0\TSRR0002_A-08_A1p_P2-A-6_01_53106.d	0.775760877	34	BL_8
X:\2019-12-05_Schaeberle_A1p_10k_SN0\TSRR0002_C-06_A1p_P2-C-6_01_53125.d	0.571533934	35	KOL_2
X:\2019-12-05_Schaeberle_A1p_10k_SN0\TSRR0002_A-11_A1p_P2-A-9_01_53109.d	0.02062157	36	BL_11
X:\2019-12-05_Schaeberle_A1p_10k_SN0\TSRR0002_D-12_A1p_P2-E-4_01_53143.d	0.005422886	37	PANIKI_4
X:\2019-12-05_Schaeberle_A1p_10k_SN0\TSRR0002_B-07_A1p_P2-B-6_01_53115.d	0.620588253	38	KM_4
X:\2019-12-05_Schaeberle_A1p_10k_SN0\TSRR0002_H-04_A1p_P2-B-9_01_53180.d	0.00171338	39	ULU_13
X:\2019-12-05_Schaeberle_A1p_10k_SN0\TSRR0002_D-07_A1p_P2-D-8_01_53137.d	0.832556165	39	KOL_18
X:\2019-12-05_Schaeberle_A1p_10k_SN0\TSRR0002_D-06_A1p_P2-D-7_01_53136.d	0.757141862	39	KOL_16
X:\2019-12-05_Schaeberle_A1p_10k_SN0\TSRR0002_C-10_A1p_P2-D-1_01_53129.d	0.728148087	39	KOL_8
X:\2019-12-05_Schaeberle_A1p_10k_SN0\TSRR0002_A-04_A1p_P2-A-2_01_53102.d	0	40	BL_2
X:\2019-12-05_Schaeberle_A1p_10k_SN0\TSRR0002_E-09_A1p_P2-F-2_01_53154.d	0	41	PANIKI_13
X:\2019-12-05_Schaeberle_A1p_10k_SN0\TSRR0002_A-05_A1p_P2-A-3_01_53103.d	0.920690168	41	BL_3
X:\2019-12-05_Schaeberle_A1p_10k_SN0\TSRR0002_A-06_A1p_P2-A-4_01_53104.d	0.034280319	42	BL_6
X:\2019-12-05_Schaeberle_A1p_10k_SN0\TSRR0002_B-03_A1p_P2-B-2_01_53111.d	0	43	BL_14
X:\2019-12-05_Schaeberle_A1p_10k_SN0\TSRR0002_F-05_A1p_P2-F-8_01_53160.d	0.609542211	44	PEHE_1
X:\2019-12-05_Schaeberle_A1p_10k_SN0\TSRR0002_A-07_A1p_P2-A-5_01_53105.d	0.712116419	44	BL_7
X:\2019-12-05_Schaeberle_A1p_10k_SN0\TSRR0002_D-08_A1p_P2-D-9_01_53138.d	0.01357126	45	KOL_21
X:\2019-12-05_Schaeberle_A1p_10k_SN0\TSRR0002_B-08_A1p_P2-B-7_01_53116.d	0.833021679	45	KM_5

Table S2. Morphological description of sponges KOL_08, KOL_16, KOL_18 and ULU_13 and underwater documentation

Code	Morphological Features	Image	Type of Spicule	Ref
KOL_8	Massive orange sponge. Hard, compressible with seemingly corrugated surface and acanthostyle typed spicule. Collected at 4 m growing on rubble in Kolongan's coral reef Sangihe Island, Indonesia. Morphological and spicule characteristics suggest assignment to *Agelas nakamurai*. It is likely that this sponge was newly grown *A. nakamurai*.	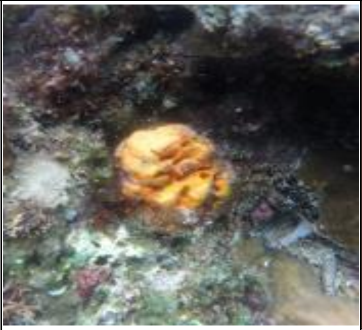 *Agelas nakamurai*	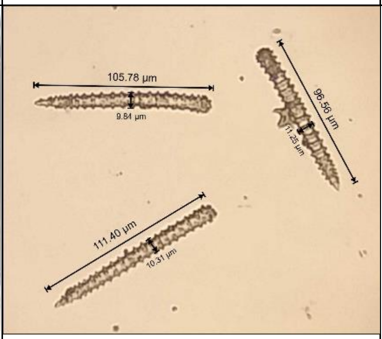 Type of spicule megascleres acanthostyle (96.56x11.25-111.40x10.31) µm.	[1]
KOL_16	Thick encrusting orange sponge. Soft, compressible with bumpy surface and acanthostyle typed spicule. Collected at ~5 m growing on corals from Kolongan's coral reef Sangihe Island, Indonesia. Morphological and spicule characteristics suggest assignment to *Agelas nakamurai*.	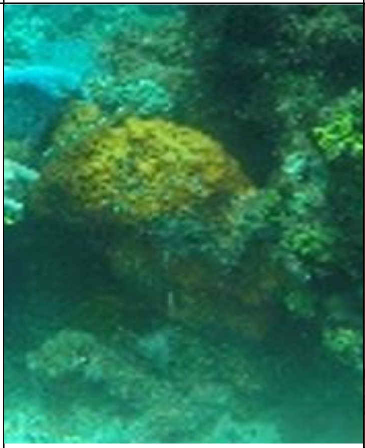 *Agelas nakamurai*	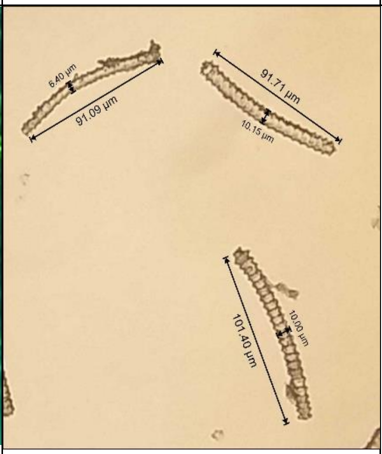 Type of spicule slightly bent megascleres acanthostyle (91.09x6.40-101.40) µm	[1]

KOL_18	Thick encrusting orange sponge. Compressible, cavernous with megascleres acanthostyle typed spicule. Collected at ~4 m growing on corals in Kolongan beach Sangihe Island. Morphological and spicule characteristics suggest assignment to *Agelas nakamurai*.	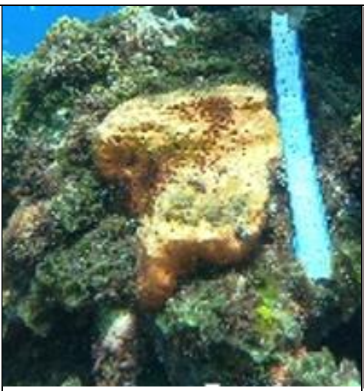 *Agelas nakamurai*	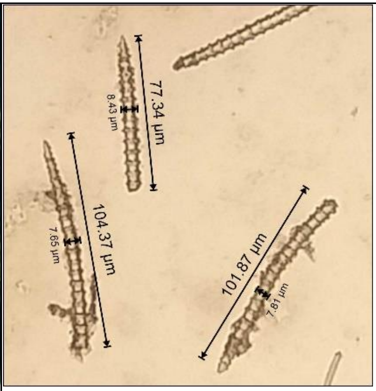 Type of spicule megascleres acanthostyle (77.34x8.34-104.37x7.65) µm.	[1]
ULU_13	Thick encrusting orange sponge. Soft, compressible (resilient) with bumpy surface, coanosome of 0.4-1.0 cm in diameter and acanthostyle typed spicule similar to KOL_8 and KOL_18 coded sponges. Collected at ~9 m from Ulu seaport, Siau Island, Indonesia. Morphological and spicule characteristics as well as comparison with those for KOL_8, and KOL_18 suggest assignment to *Agelas nakamurai*.	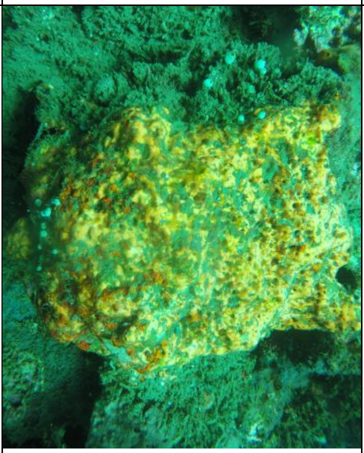 *Agelas nakamurai*	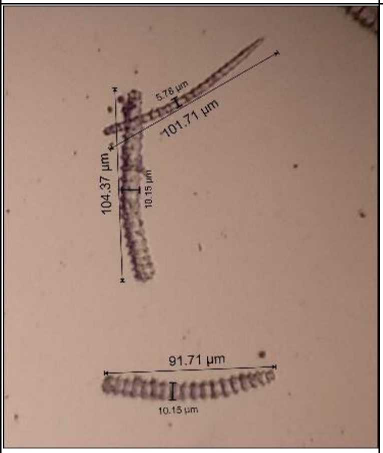 Type of spicule megascleres acanthostyle wide (91.71x10.15-104.37x10.15) µm and thin (101.71x5.78) µm	[1]

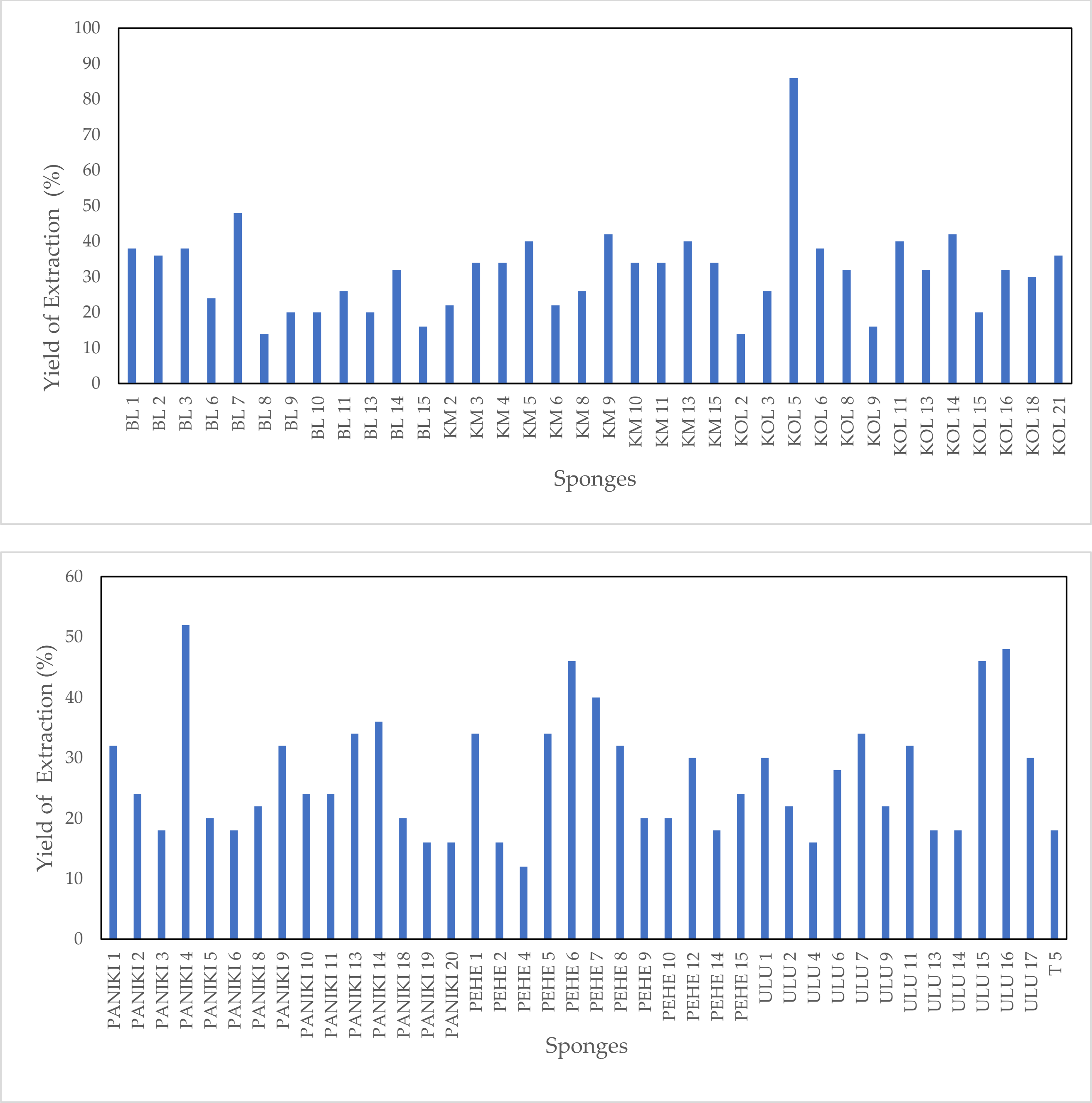

Figure S1. Sponges investigated in this study and extraction yield

	1	2	3	4	5	6	7	8	9	10	11	12	13	14	15	16	17	18	19	20	21	22	23	24
A	100	100	100	22	14	17	14	21	21	18	18	5	17	17	10	6	5	14	-12	15	2	-7	-2	-6
B	100	102	102	27	-10	31	15	20	19	10	40	14	20	13	26	9	4	16	28	5	-1	-7	-9	-39
C	100	101	100	24	2	-1	10	-4	0	3	0	-11	-8	-14	-20	-18	-24	-20	-45	-44	-41	-23	-35	-9
D	101	103	103	-8	100	-3	-17	3	9	-23	-17	-3	-5	-17	-20	-21	-3	-38	-24	-20	-16	-33	-42	-42
E	100	100	101	14	100	-3	100	100	4	6	4	-23	3	-7	-12	-14	-9	5	-5	-6	-5	-35	-39	-15
F	97	102	102	-6	1	-37	-24	-12	20	-11	-6	-5	18	-11	3	1	3	-21	-10	-20	-8	-35	-19	-50
G	100	100	99	-3	8	-9	7	0	-28	12	-11	-1	10	-1	-24	-5	-24	-24	-24	-30	-38	-34	-37	-40
H	100	97	98	-14	-2	7	-13	-2	-16	22	-13	21	13	-4	11	13	-11	10	1	-2	-10	-14	-17	-48
I	100	101	101	-6	-4	-31	-31	-39	-37	-37	-42	-45	-45	-48	-50	-62	-60	-60	-61	-66	-69	-83	-76	-61
J	100	13	11	5	-21	-27	-18	-9	-15	-13	-26	-15	-18	-12	-41	-26	-33	-31	-34	-24	-53	-55	-85	-63
K	97	-11	-11	-8	-37	-11	-17	-29	-49	-42	-52	-29	-49	-44	-53	-46	-55	-40	-56	-50	-61	-40	-70	-71
L	101	7	4	-7	102	-7	-5	-14	-6	-10	-13	-10	-14	-3	-30	-16	-11	-14	-21	-21	-33	-36	-41	-42
M	100	-11	-10	-12	101	-19	101	102	-31	-26	-44	-37	-27	-20	-38	-28	-21	-16	-26	-24	-31	-24	-35	-53
N	101	-15	-7	-15	-18	-18	-13	-1	-36	-22	-17	-26	-20	-26	-17	-30	-35	-29	-37	-34	-40	-40	-42	-53
O	101	-9	-18	3	-15	1	-9	-12	-12	-23	-14	-15	-9	-19	-19	-8	-46	-42	-16	-24	-23	-35	-39	-53
P	102	-7	-6	-16	-79	-3	-13	-10	-26	-24	-35	-12	-31	-27	-20	-25	-15	-7	-33	-16	-21	-39	-45	-7

(a)

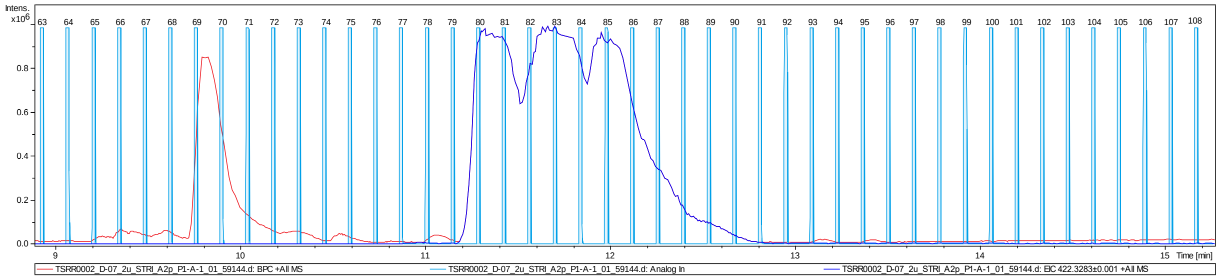

(b)

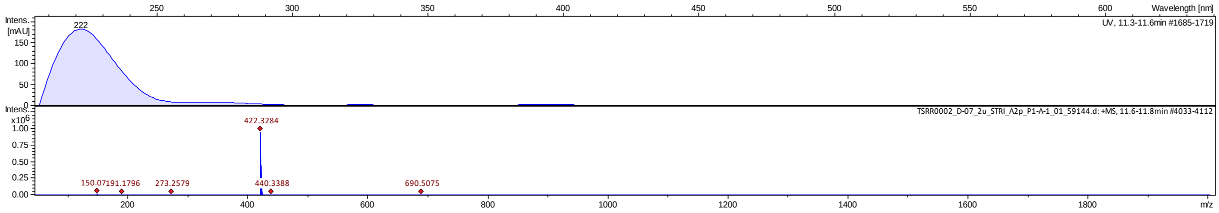

(c)

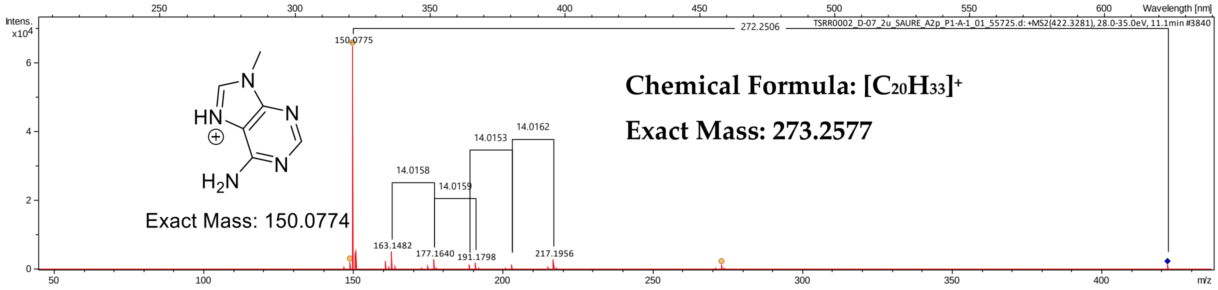

(d)

Figure S2. Microfractionation: Bioassay and dereplication results plate KOL_18 against *S. aureus* (a) Assay read-out of microfractionation plate KOL_18 against *S. aureus*. Numbers indicate the relative growth inhibition of the test strain. Fractions causing at least 70% rel. growth inhibition were considered "active". Column 1: Medium control; Columns 2 + 3 antibiotic standard (Gentamycin 64 - 0.002 µg/mL); Column 4: Growth control. Area AH05- AH24: 1 µL injection volume replicate showing activity in fractions 80-81 and 83-84; Area IP05- IP24: 2 µL injection volume replicate showing activity in fractions 80-81 and 83-84; **(b)** Overlaid base peak chromatogram (red), fraction collector analog signal (light blue bars) and extracted ion chromatogram of *m/z* 422.3281 ± 0.001 $[M]^+$ (dark blue) of the 1 mg/mL (in MeOH) with 2 µL injection volume; **(c)** UV chromatogram and MS spectrum of fractions 80-84; **(d)** MS/MS fragmentation of the precursor ion at *m/z* 422.3281 $[M]^+$ (dereplicated as agelasine A-F), manual annotation of the neutral losses and proposed structure of the base peak ion.

	1	2	3	4	5	6	7	8	9	10	11	12	13	14	15	16	17	18	19	20	21	22	23	24
A	103	110	109	16	-16	-12	-13	-28	-39	-5	-17	-28	-26	-43	-33	22	-21	-27	-42	-48	-38	-31	-18	-6
B	102	110	109	20	13	-5	-5	-17	-3	-16	9	0	-14	-7	-29	-31	-21	-34	-28	-32	-26	-30	-29	-14
C	102	107	105	-37	-30	-38	-37	-20	-24	-16	-34	-50	-30	-53	-58	-27	-52	-39	-36	-73	-44	-44	-17	-10
D	94	20	34	25	106	22	21	14	25	16	-12	-12	9	16	6	-11	-19	-37	-41	-47	-50	-46	-29	-12
E	101	109	108	4	106	104	107	19	76	24	-4	-12	-1	-43	-34	-28	-32	-28	-30	-57	-55	-43	-29	-23
F	101	107	108	4	-7	6	5	11	10	-24	-10	-18	7	-13	-22	-40	-36	-46	-32	-32	-42	-48	-46	-31
G	100	-33	104	14	-28	-15	-30	-17	-12	-19	-27	-20	-27	-25	-35	-33	-45	-44	-55	-47	-66	-40	-28	-17
H	98	-119	32	8	105	3	2	4	-7	-5	-19	-3	-26	-25	-19	-5	-11	-32	-47	-34	-20	-26	-11	-29
I	99	-102	-9	-9	-55	-45	-51	-65	-37	-54	-43	-50	-59	-48	-65	-88	-65	-63	-56	-47	-48	-63	-44	-34
J	98	-107	-16	7	9	3	6	-1	2	-16	-6	-63	-60	-31	-21	-19	-41	-20	-28	-36	-43	-33	-36	-11
K	99	-31	-17	-16	-40	-34	-20	-34	-27	-24	-20	-13	-18	-11	-28	-15	-42	-60	-61	-65	-49	-52	-16	-27
L	100	-111	-13	9	107	108	97	-14	-10	3	-7	-14	-14	21	17	94	-9	-19	-16	-44	-44	-28	-24	-8
M	99	-35	-39	-34	107	109	109	107	107	101	10	4	2	-20	-31	-46	-41	-35	-51	-31	-34	-25	-32	-1
N	101	-15	-9	-13	-19	-1	-2	-16	-16	0	-25	-1	9	4	-7	-14	-15	-52	-21	-24	-32	-15	-14	0
O	100	-18	-26	3	-17	-46	-10	-17	-18	-11	-30	-15	-4	-9	-19	-7	-23	-13	-33	-8	-21	-17	-21	-17
P	101	19	-1	-1	106	-6	-6	5	-8	10	7	3	6	16	4	1	12	3	0	21	7	1	-2	-7

(a)

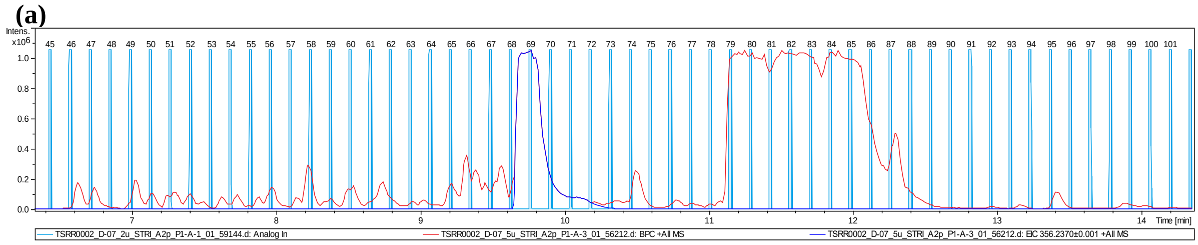

(b)

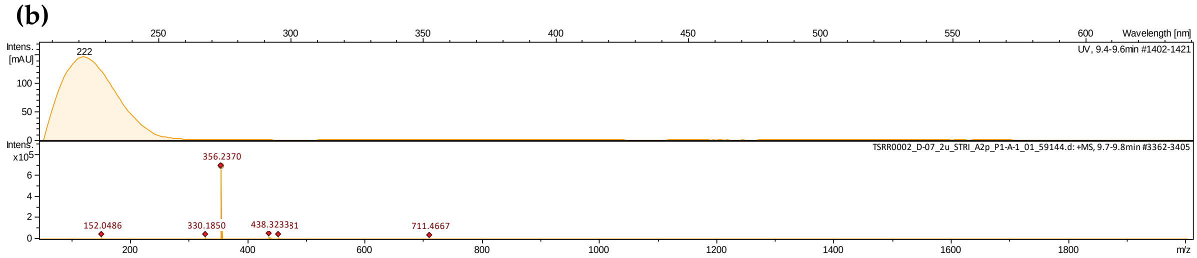

(c)

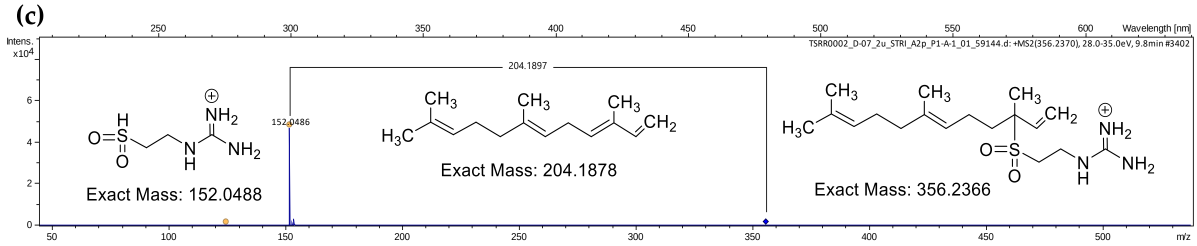

(d)

Figure S3. Microfractionation: Bioassay and dereplication results plate KOL_18 against *S. triciti* (a) Assay read-out of the microfractionation plate KOL_18 against *S. triciti*. Numbers indicate the relative growth inhibition of the test strain. Fractions causing at least 70% rel. growth inhibition were considered “active”. Column 1: Medium control; Columns 2 + 3 antibiotic standard (Nystatin 25.6 – 0.0008 µg/mL); Column 4: Growth control. Area AH05- AH24: 2 µL injection volume replicate showing activity in fractions 80-83 and 85; Area IP05- IP24: 5 µL injection volume replicate showing activity in fractions 69 and 80-85; H5 and P5: unfractionated extract control **(b)** Overlaid base peak chromatogram (red), fraction collector analog signal (light blue bars) and extracted ion chromatogram of *m*/*z* 355.2370 ± 0.001 $[M+H]^+$ (dark blue) of the 1 mg/mL (in MeOH) with 5 µL injection volume; **(c)** UV chromatogram and MS spectrum of fraction 69; **(d)** MS/MS fragmentation of the precursor ion at *m*/*z* 356.2370 $[M+H]^+$ (dereplicated as agelasidine A), manual annotation of the neutral loss and proposed structure of the base peak ion.

	1	2	3	4	5	6	7	8	9	10	11	12	13	14	15	16	17	18	19	20	21	22	23	24
A	101	101	99	28	24	29	15	34	32	25	41	37	23	30	8	19	32	47	5	38	30	36	37	44
B	101	101	100	7	2	14	-48	21	10	24	26	23	-2	23	8	36	22	20	34	14	-4	36	25	52
C	101	101	99	-11	18	16	6	30	30	32	19	16	18	-4	30	29	26	17	33	1	-12	30	22	29
D	101	101	99	-1	15	8	-17	25	2	8	26	6	51	82	25	25	12	18	29	16	8	32	31	-20
E	101	99	88	-7	0	96	95	81	92	97	-3	96	-58	18	14	21	17	12	35	28	23	17	21	37
F	100	27	25	-4	14	7	19	22	-5	2	13	16	5	7	23	16	27	31	43	23	8	-3	22	34
G	100	-4	19	-4	2	16	16	2	14	18	0	10	10	0	4	21	15	14	33	-3	12	-10	31	25
H	100	-6	11	4	96	0	15	-1	22	-1	10	3	16	7	10	8	24	-1	13	14	10	20	27	29
I	100	9	-15	3	2	4	14	21	4	14	18	5	17	4	20	21	23	21	33	-1	22	35	-4	27
J	100	7	6	9	12	-3	26	12	-2	5	17	25	-2	16	19	20	5	17	30	19	17	32	-17	35
K	100	2	16	-12	-6	20	2	-8	-3	2	8	-7	-3	-2	-1	11	-3	13	14	1	26	27	-11	24
L	99	-11	-9	0	96	0	-12	-6	-14	6	12	12	36	97	97	0	-15	15	21	11	-30	15	5	21
M	100	-4	4	12	77	80	97	98	98	98	99	97	-37	8	9	4	-11	6	15	13	6	5	11	16
N	99	-13	5	-24	-13	-5	-22	-2	25	2	8	4	15	11	7	13	8	3	23	20	-30	22	6	22
O	100	4	2	5	4	-7	8	4	-19	6	3	-3	-23	2	-10	-14	1	5	2	18	6	5	11	27
P	100	17	17	-6	92	24	11	26	-21	10	8	1	9	11	9	19	-11	-4	25	33	8	9	7	23

(a)

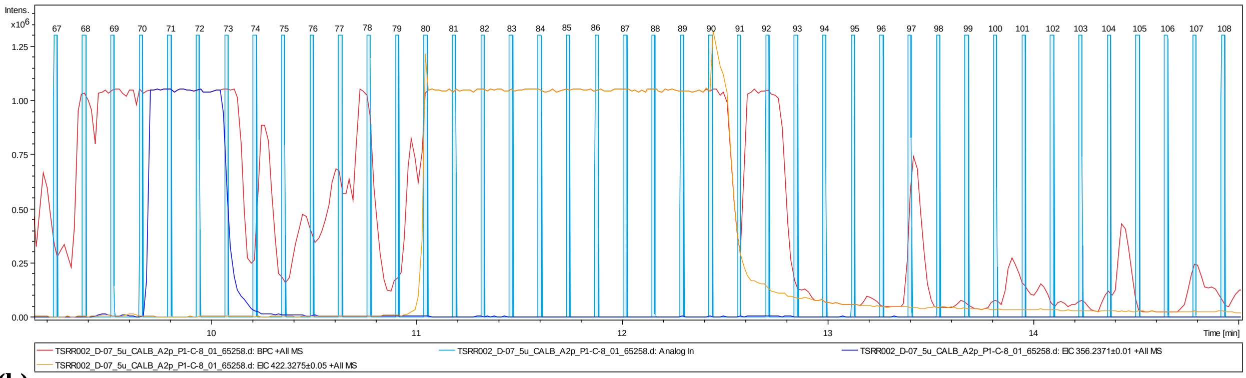

(b)

Figure S4. Microfractionation: Bioassay and dereplication results plate KOL_18 against *C. albicans* (a) Assay read-out of the microfractionation plate KOL_18 against *C. albicans*. Numbers indicate the relative growth inhibition of the test strain. Fractions causing at least 70% rel. growth inhibition were considered "active". Column 1: Medium control; Columns 2 + 3 antibiotic standard (Nystatin 64 - 0.002 µg/mL); Column 4: Growth control. Area AH05- AH24: 2 µL injection volume replicate showing activity in fractions 71, 82-86 and 88; Area IP05- IP24: 5 µL injection volume replicate showing activity in fractions 70-71, 80 and 83-88; H5 and P5: Unfractionated extract control **(b)** Overlaid base peak chromatogram (red), fraction collector analog signal (light blue bars), extracted ion chromatogram of *m*/*z* 355.2370 ± 0.001 [M+H]$^+$ (dark blue)-dereplicated as agelasidine A and extracted ion chromatogram of *m*/*z* 422.3281 ± 0.005 [M]$^+$ (orange)-dereplicated as agelasine (A-F) of the 2 mg/mL (in MeOH) with 5 µL injection volume.

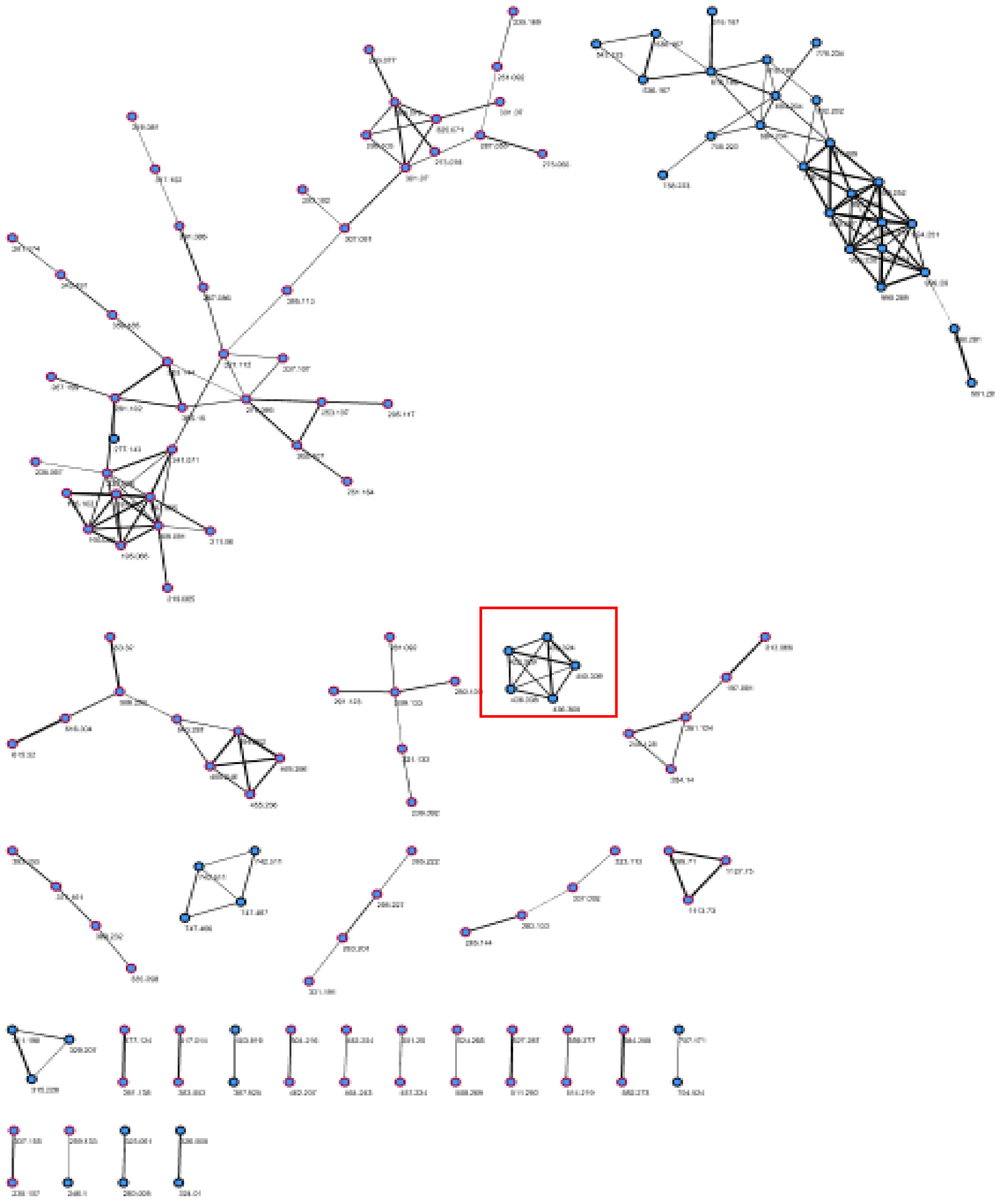

Figure S5. Molecular networking of the analyzed sponge sample set. The cluster of the agelasine family is framed in red.

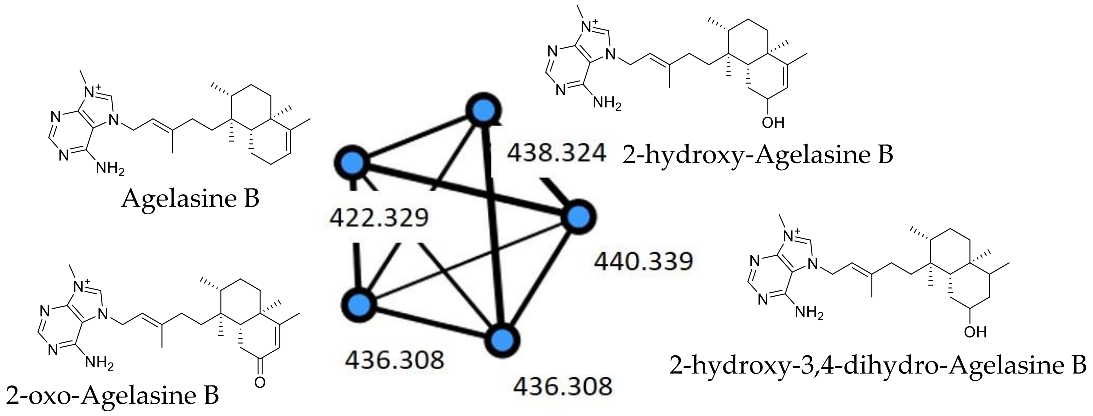

(a)

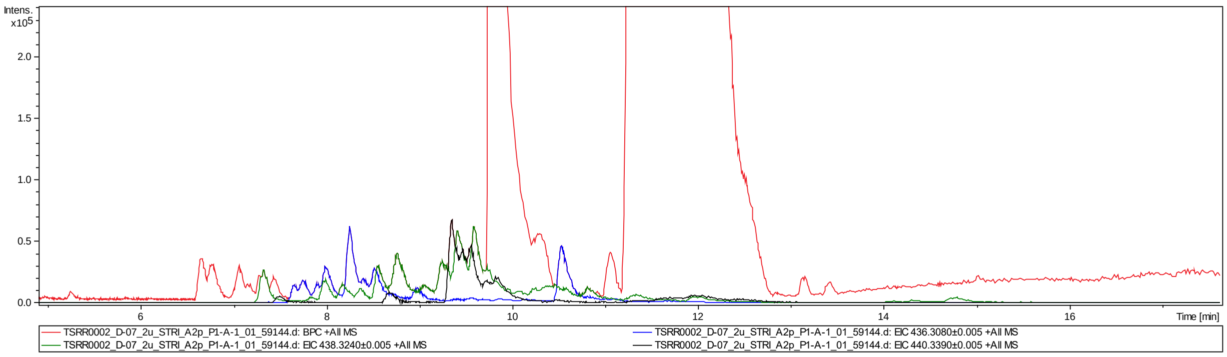

(b)

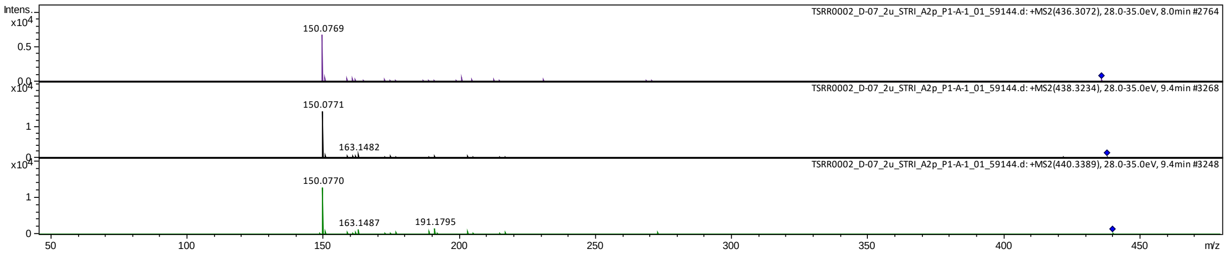

(c)

Figure S6. Enlarged cluster of agelasine derivatives and MS/MS fragmentation analysis (a) Cluster of agelasine derivatives present in the extract of KOL_18 with selected structures corresponding to the precursor ions (framework of agelasine B was chosen as example); **(b)** Overlaid base peak chromatogram (red) of the KOL_18 extract, extracted ion chromatogram, of *m*/*z* 436.3080 ± 0.005 (blue), *m*/*z* 438.3240 ± 0.005 (green) and *m*/*z* 440.3390 ± 0.005 (black); **(c)** MS/MS fragmentation of the selected precursor ions.

	1	2	3	4	5	6	7	8	9	10	11	12	13	14	15	16	17	18	19	20	21	22	23	24
A	100	100	100	21	21	16	17	19	22	21	25	18	14	22	10	5	3	2	0	1	-6	5	13	6
B	100	101	101	25	-5	4	6	12	8	10	7	-3	6	2	7	17	18	16	-3	29	23	12	3	-21
C	100	100	100	6	2	2	2	7	0	1	-9	-14	-11	-13	-25	-29	-19	-16	-32	-32	-36	-22	-35	-15
D	100	100	100	9	3	-28	-16	11	4	-24	-10	1	-16	-20	3	-8	-3	-12	-3	2	-7	1	-31	20
E	100	100	100	-1	16	8	12	12	16	10	8	3	1	-4	-17	-15	-45	-18	-24	-35	-32	-44	-50	-42
F	100	101	101	5	-5	20	7	7	1	-2	10	2	5	-6	-16	-3	-50	-9	-17	-17	-22	-24	-18	-42
G	100	99	68	-1	14	3	15	5	4	14	5	1	11	5	-20	7	5	3	-2	-5	-6	-6	-8	-24
H	100	100	100	-5	-6	-1	0	-3	26	4	4	4	11	1	4	23	-1	-2	1	-21	6	-18	-6	-27
I	99	100	101	1	10	-11	-33	-42	-30	-28	-37	-42	-41	-41	-49	-46	-56	-50	-58	-47	-57	-59	-48	-59
J	100	48	83	-6	-18	-17	-9	1	-16	-8	-6	-7	-13	-8	-16	-8	-18	-22	-25	-27	-20	-33	-36	-39
K	100	9	23	-6	-10	-32	-26	-29	-28	-29	-20	85	79	-41	-46	-37	-50	-50	-49	-35	-58	-53	-50	-58
L	100	0	-4	-3	-12	-8	-10	-9	-9	-12	-10	-12	-11	-4	-14	-15	-20	-19	-21	-21	-16	-24	-21	-39
M	100	2	-5	-21	-16	-14	-15	-27	-33	-20	-18	-32	-25	-22	-26	-37	-41	-28	-40	-32	-25	-23	-26	-42
N	101	2	5	-14	-14	-15	-14	-6	-24	-19	-11	-14	-11	-18	-19	-38	91	-23	-16	-20	-22	-24	-19	-27
O	100	24	11	9	-17	3	5	-6	-7	-13	-14	-12	-29	-18	-29	-28	-17	-14	-9	-15	-16	-15	-8	-12
P	101	-9	54	-19	-16	-11	-8	-8	-19	-11	-12	-21	-9	-13	-17	-17	-9	-5	-14	-10	0	-4	-3	-3

(a)

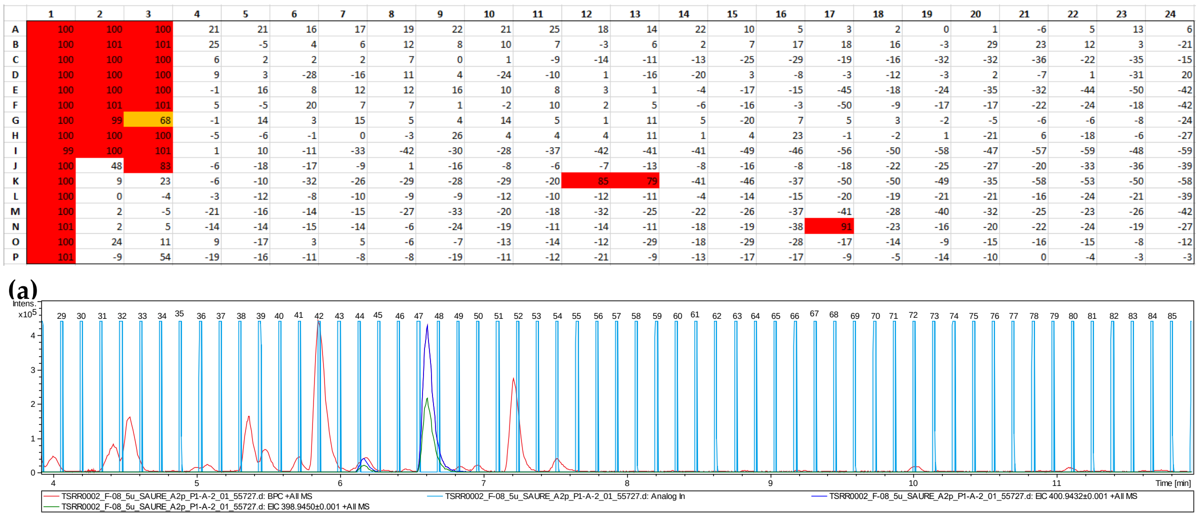

(b)

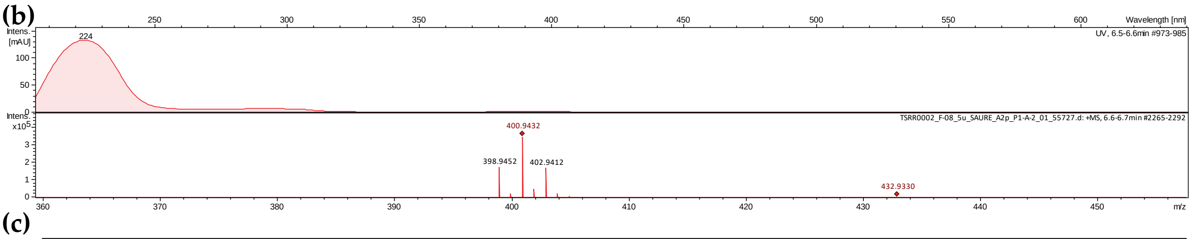

(c)

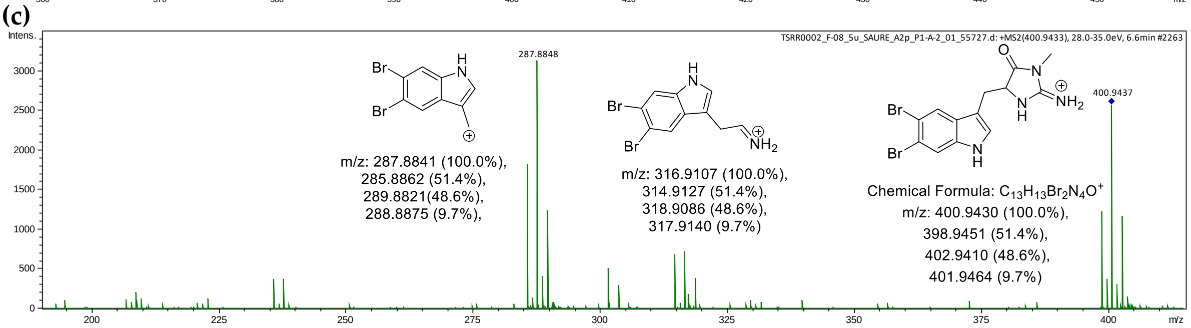

(d)

Figure S7. Microfractionation: Bioassay and dereplication results plate PEHE_5 against *S. aureus* fractions 47-48 (a) Assay read-out of microfractionation plate PEHE_5 against *S. aureus*. Numbers indicate the relative growth inhibition of the test strain. Fractions causing at least 70% rel. growth inhibition were considered "active". Column 1: Medium control; Columns 2 + 3 antibiotic standard; Column 4: Growth control. Area AH05-AH24: 2µL injection volume; Area IP05- IP24 : 5 µL injection; **(b)** Overlaid base peak chromatogram (red), fraction collector analog signal (light blue bars) and extracted ion chromatogram of *m/z* 398.9450 ± 0.001 $[M+H]^+$ (dark blue) and 400.9432 ± 0.001 $[M+H]^+$ (green) of the 1 mg/mL (in MeOH) with 5 µL injection volume; **(c)** UV chromatogram and MS spectrum of fractions 47-48; **(d)** MS/MS fragmentation of the precursor ion at *m/z* 400.9432 $[M+H]^+$ (dereplicated as 5,6-dibromo-1′,8-dihydro-2′-demethylaplysinopsin), and proposed structures of the fragment ions.

	1	2	3	4	5	6	7	8	9	10	11	12	13	14	15	16	17	18	19	20	21	22	23	24
A	100	100	100	21	21	16	17	19	22	21	25	18	14	22	10	5	3	2	0	1	-6	5	13	6
B	100	101	101	25	-5	4	6	12	8	10	7	-3	6	2	7	17	18	16	-3	29	23	12	3	-21
C	100	100	100	6	2	2	2	7	0	1	-9	-14	-11	-13	-25	-29	-19	-16	-32	-32	-36	-22	-35	-15
D	100	100	100	9	3	-28	-16	11	4	-24	-10	1	-16	-20	3	-8	-3	-12	-3	2	-7	1	-31	20
E	100	100	100	-1	16	8	12	12	16	10	8	3	1	-4	-17	-15	-45	-18	-24	-35	-32	-44	-50	-42
F	100	101	101	5	-5	20	7	7	1	-2	10	2	5	-6	-16	-3	-50	-9	-17	-17	-22	-24	-18	-42
G	100	99	68	-1	14	3	15	5	4	14	5	1	11	5	-20	7	5	3	-2	-5	-6	-6	-8	-24
H	100	100	100	-5	-6	-1	0	-3	26	4	4	4	11	1	4	23	-1	-2	1	-21	6	-18	-6	-27
I	99	100	101	1	10	-11	-33	-42	-30	-28	-37	-42	-41	-41	-49	-46	-56	-50	-58	-47	-57	-59	-48	-59
J	100	48	83	-6	-18	-17	-9	1	-16	-8	-6	-7	-13	-8	-16	-8	-18	-22	-25	-27	-20	-33	-36	-39
K	100	9	23	-6	-10	-32	-26	-29	-28	-29	-20	85	79	-41	-46	-37	-50	-50	-49	-35	-58	-53	-50	-58
L	100	0	-4	-3	-12	-8	-10	-9	-9	-12	-10	-12	-11	-4	-14	-15	-20	-19	-21	-21	-16	-24	-21	-39
M	100	2	-5	-21	-16	-14	-15	-27	-33	-20	-18	-32	-25	-22	-26	-37	-41	-28	-40	-32	-25	-23	-26	-42
N	101	2	5	-14	-14	-15	-14	-6	-24	-19	-11	-14	-11	-18	-19	-38	91	-23	-16	-20	-22	-24	-19	-27
O	100	24	11	9	-17	3	5	-6	-7	-13	-14	-12	-29	-18	-29	-28	-17	-14	-9	-15	-16	-15	-8	-12
P	101	-9	54	-19	-16	-11	-8	-8	-19	-11	-12	-21	-9	-13	-17	-17	-9	-5	-14	-10	0	-4	-3	-3

(a)

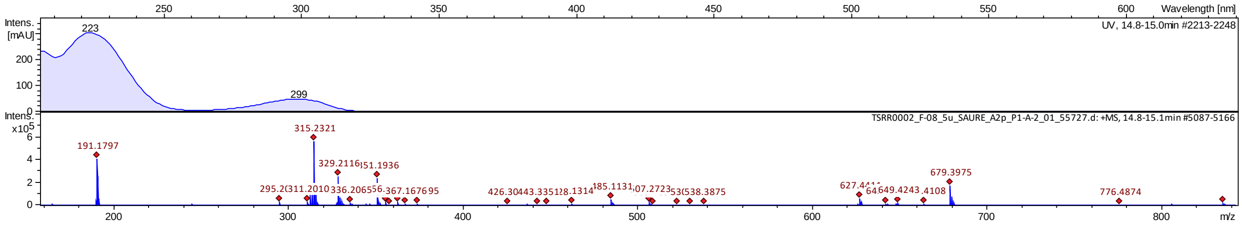

(b)

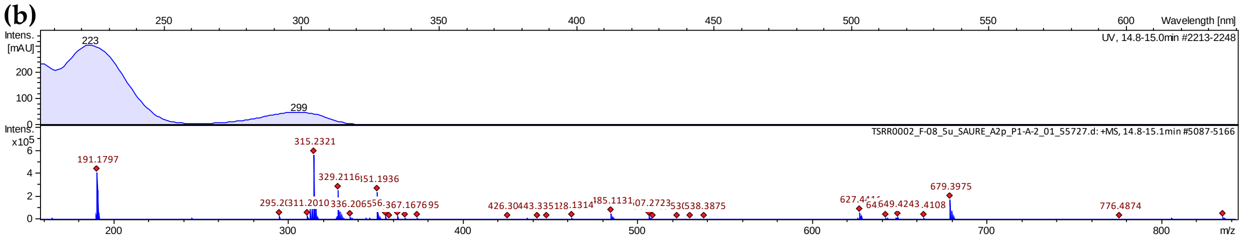

(c)

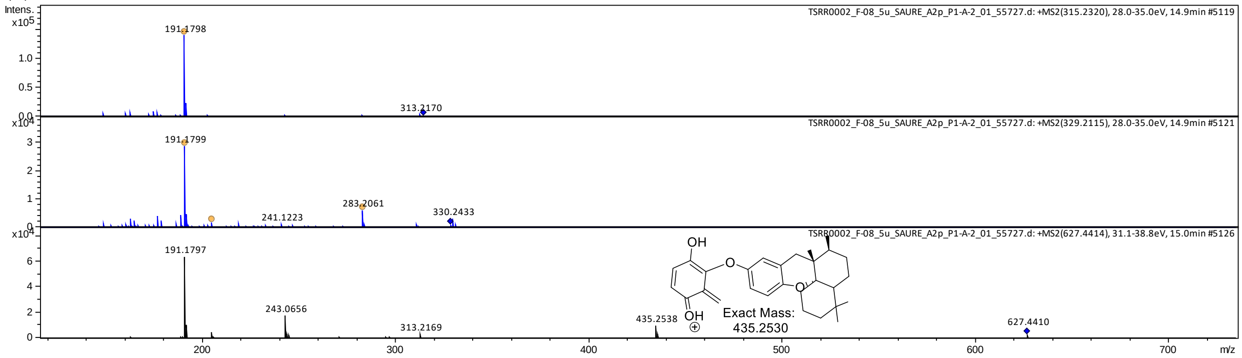

Figure S8. Microfractionation: Bioassay and dereplication results plate PEHE_5 against *S. aureus* fraction 108 **(a)** Assay read-out of µ-fractionation plate PEHE_5 against *S. aureus*. Numbers indicate the relative growth inhibition of the test strain. Fractions causing at least 70% rel. growth inhibition were considered "active". Column 1: Medium control; Columns 2 + 3 antibiotic standard; Column 4: Growth control. Area AH05- AH24: 2 µL injection volume; Area IP05- IP24 : 5 µL injection; **(b)** Overlaid base peak chromatogram (red), fraction collector analog signal (light blue bars) and extracted ion chromatogram of *m/z* 315.2321±0.001 [M+H]+ (dark blue), *m/z* 329.2115 ± 0.001 [M+H]+ (green) and *m/z* 627.4414 [M+H]+ of the 1 mg/mL (in MeOH) with 5 µL injection volume; **(c)** UV chromatogram and MS spectrum of fraction 108; **(d)** MS/MS fragmentation of the precursor ions at *m/z* 315.2321 [M+H]+, *m/z* 329.2115 [M+H]+ and *m/z* 627.4414 [M+H]+, tentatively assigned to the group of sesquiterpene hydroquinones.

	1	2	3	4	5	6	7	8	9	10	11	12	13	14	15	16	17	18	19	20	21	22	23	24
A	102	110	110	13	9	-3	3	-8	9	-9	-6	-15	-4	7	11	-2	14	-23	4	-1	-4	-18	-5	-22
B	99	110	109	-8	0	-34	-18	5	28	59	99	100	63	53	49	9	12	17	-4	5	10	-17	-10	9
C	102	110	109	2	6	8	11	1	13	1	1	6	13	7	16	10	18	-5	-6	-26	-22	-17	-31	-17
D	n.a.	109	109	-13	-23	-1	-2	-22	-9	-19	-19	-4	8	0	-13	-33	-12	-18	-14	-15	-8	-42	-18	23
E	99	110	109	7	19	16	4	26	24	11	-4	15	13	20	4	5	-14	-15	-25	-8	-34	-19	-16	-21
F	98	109	107	-30	-25	-3	-13	-1	-3	9	5	14	1	-2	-3	-4	-6	2	-4	-2	6	7	-3	-6
G	102	102	89	10	15	23	13	15	15	7	1	16	9	3	16	12	2	9	6	13	-5	-14	-14	-11
H	99	-17	-9	-19	103	4	1	-6	-3	-3	-1	-2	-7	2	9	8	12	4	-7	11	1	-11	0	-17
I	103	17	14	7	-2	4	-1	-3	3	-2	5	4	1	13	3	15	22	26	-5	5	-1	17	14	-12
J	97	1	3	-4	13	20	14	34	69	97	106	107	103	94	84	77	59	56	35	20	16	11	-1	8
K	105	11	10	-1	5	7	6	25	33	27	25	18	15	21	8	23	24	19	3	7	14	0	-17	-17
L	97	-4	-14	7	15	4	-3	12	-2	12	16	23	18	12	-17	10	10	15	-5	-10	-9	-4	-29	2
M	102	18	23	8	14	-3	14	4	1	7	8	11	8	7	4	18	24	7	-13	3	33	-1	-1	4
N	98	0	-3	7	5	-2	-1	9	0	7	6	-4	18	-7	20	14	2	7	-7	12	3	-10	-14	-4
O	100	21	24	19	22	10	16	16	16	9	-6	14	3	22	19	24	16	18	10	13	8	-4	0	-47
P	97	-7	10	-7	107	-2	3	3	14	5	3	-5	5	5	-2	-3	5	12	7	-10	-1	8	-4	-1

(a)

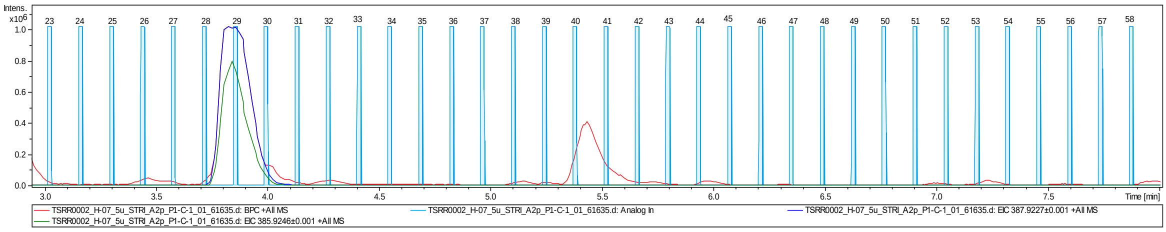

(b)

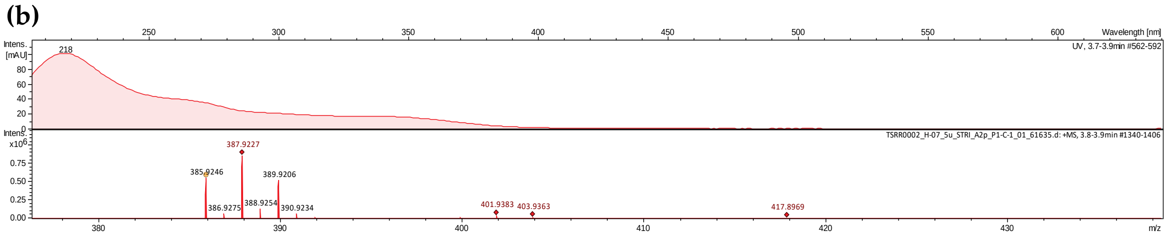

(c)

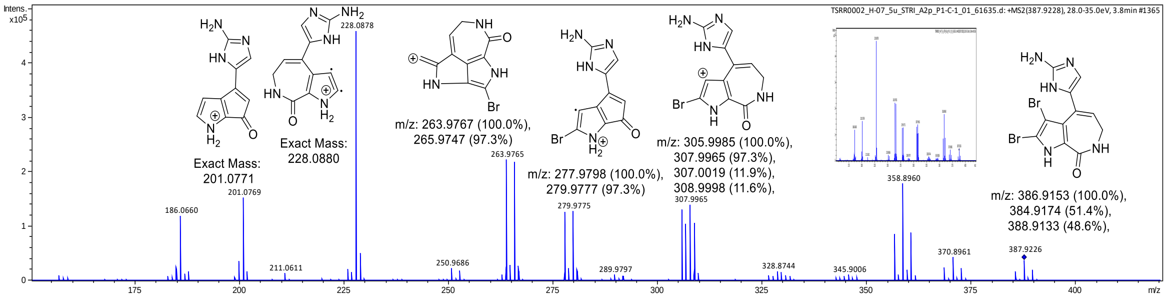

(d)

Figure S9. Microfractionation: Bioassay and dereplication results plate ULU_16 against *S. triciti* (a) Assay read-out of µ-fractionation plate ULU_16 against *S. triciti*. Numbers indicate the relative growth inhibition of the test strain. Fractions causing at least 70% rel. growth inhibition were considered "active". Column 1: Medium control; Column 2 + 3 antibiotic standard; Column 4: growth control. Area AH05- AH24: 2µL injection volume; Area IP05- IP24 : 5 µL injection; H5 and P5 unfractionated extract control **(b)** Overlaid base peak chromatogram (red), fraction collector analog signal (light blue bars) and extracted ion chromatogram of *m/z* 385.9249 ± 0.001 $[M+H]^+$ (green) and 387.9227 ± 0.001 $[M+H]^+$ (dark blue) of the 1 mg/mL (in MeOH) with 5 µL injection volume; **(c)** UV chromatogram and MS spectrum of fractions 29-30; **(d)** MS/MS fragmentation of the precursor ion at *m/z* 387.9227 $[M+H]^+$ (dereplicated as stevensine/odiline), and proposed structures of the fragment ions.

	1	2	3	4	5	6	7	8	9	10	11	12
A	104	104	103	37	-20	-20	-12	1	-11	-13	1	4
B	96	104	103	13	-2	-5	-11	5	-13	6	-6	-13
C	104	104	102	16	18	-30	-43	-18	-47	-17	-18	-33
D	n.a.	102	37	25	14	16	22	-9	5	1	-29	-30
E	104	104	100	19	14	36	-10	-29	-18	-17	-19	-27
F	97	103	51	-13	-48	-25	-33	-21	-9	-9	-11	-27
G	104	103	3	10	2	-28	-1343	-52	-40	-46	-20	-20
H	96	91	-93	-5	52	2	-33	-1	-1	-18	-26	-24
I	104	101	-86	-4	-34	-4	-65	-45	-24	-24	-49	-68
J	96	19	-93	-2	-10	-25	-17	-22	-25	-39	-33	-24
K	104	101	-30	0	-25	-16	-52	-25	-53	-49	-47	-96
L	95	-10	-118	-29	-5	-47	-40	-46	3	-28	-8	-28
M	104	101	-27	-28	17	27	-4	-59	-45	-43	-32	-52
N	95	-2	-35	-29	-46	-43	-28	-42	-54	-52	-58	-22
O	103	12	-21	-9	1	-13	-65	-22	-27	-38	-23	-7
P	95	-19	-31	-1	77	-36	4	-24	-23	-15	-3	-5

	13	14	15	16	17	18	19	20	21	22	23	24
A	-23	-14	-30	-21	-20	-41	5	-72	-14	-41	-33	-16
B	-4	-3	-10	-32	-26	-37	-43	-37	-58	-33	-42	-27
C	-33	-37	-3	3	-31	-45	-3	-49	-63	-33	-21	-23
D	-22	-30	-37	-38	-24	-56	-63	-67	-62	-32	-60	29
E	-29	-23	-6	-24	-29	-44	20	-61	-58	-53	-44	-23
F	-29	-18	-30	-28	-52	-38	-43	-35	-21	-28	-44	-31
G	-35	-46	-65	-42	-30	-37	-3	-52	-51	-46	-41	-5
H	-25	-26	-10	4	-15	-5	-11	-19	-32	-27	-42	-16
I	-68	-69	-41	-34	-49	-12	-47	-48	-29	-46	-48	-5
J	-14	-31	-22	-22	-21	-35	-38	-32	-61	-28	-27	-21
K	-71	-51	-47	-40	-45	-8	-38	-45	-48	-29	-16	-17
L	-30	-23	-29	-16	-24	-27	-30	-35	-48	-31	-34	-20
M	-38	-36	-20	-24	-13	85	83	-14	-38	-38	-21	-31
N	-118	-32	-32	-31	-24	-23	-32	-46	-6	-15	-32	-10
O	-17	-10	-12	4	-11	-31	1	-23	-15	-20	-2	-19
P	-6	-11	-2	-9	2	-3	2	-7	-13	-2	-10	-8

(a)

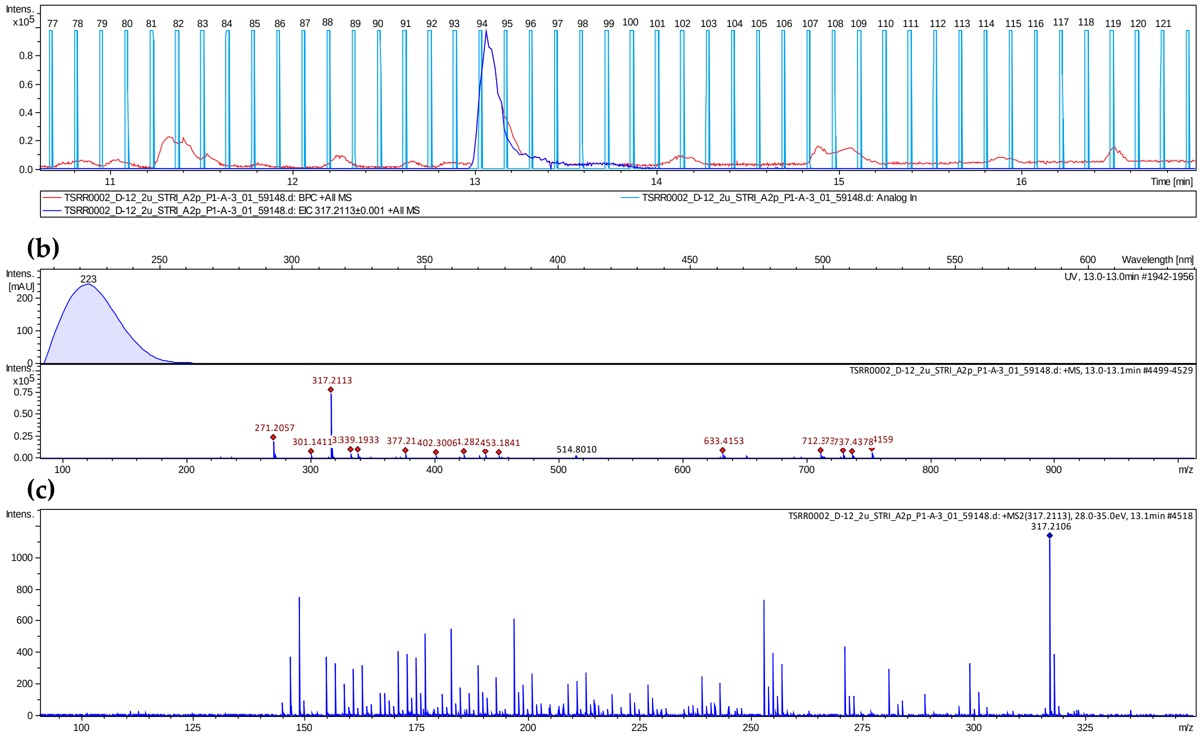

(d)

Figure S10. Microfractionation: Bioassay and dereplication results plate PANIKI_4 against *S. triciti* (a) Assay read-out of µ-fractionation plate PANIKI_4 against *S. triciti*. Numbers indicate the relative growth inhibition of the test strain. Fractions causing at least 70% rel. growth inhibition were considered "active". Column 1: Medium control; Column 2 + 3 antibiotic standard; Column 4: growth control. Area AH05- AH24: 2µL injection volume; Area IP05- IP24 : 5 µL injection; **(b)** Overlaid base peak chromatogram (red), fraction collector analog signal (light blue bars) and extracted ion chromatogram of *m/z* 317.2112 ± 0.001 [M+H]$^+$ (dark blue) of the 1 mg/mL (in MeOH) with 5 µL injection volume; **(c)** UV chromatogram and MS spectrum of fractions 94-95; **(d)** MS/MS fragmentation of the precursor ion at *m/z* 317.2112 [M+H]$^+$ (tentatively assigned the structure of 20-hydroxyhaterumadienone).

	1	2	3	4	5	6	7	8	9	10	11	12	13	14	15	16	17	18	19	20	21	22	23	24
A					1	2	3	4	5	6	7	8	9	10	11	12	13	14	15	16	17	18	19	20
B					40	39	38	37	36	35	34	33	32	31	30	29	28	27	26	25	24	23	22	21
C					41	42	43	44	45	46	47	48	49	50	51	52	53	54	55	56	57	58	59	60
D					80	79	78	77	76	75	74	73	72	71	70	69	68	67	66	65	64	63	62	61
E					81	82	83	84	85	86	87	88	89	90	91	92	93	94	95	96	97	98	99	100
F					120	119	118	117	116	115	114	113	112	111	110	109	108	107	106	105	104	103	102	101
G					121	122	123	124	125	126	127	128	129	130	131	132	133	134	135	136	137	138	139	140
H					Pos.con.	159	158	157	156	155	154	153	152	151	150	149	148	147	146	145	144	143	142	141
I					1	2	3	4	5	6	7	8	9	10	11	12	13	14	15	16	17	18	19	20
J					40	39	38	37	36	35	34	33	32	31	30	29	28	27	26	25	24	23	22	21
K					41	42	43	44	45	46	47	48	49	50	51	52	53	54	55	56	57	58	59	60
L					80	79	78	77	76	75	74	73	72	71	70	69	68	67	66	65	64	63	62	61
M					81	82	83	84	85	86	87	88	89	90	91	92	93	94	95	96	97	98	99	100
N					120	119	118	117	116	115	114	113	112	111	110	109	108	107	106	105	104	103	102	101
O					121	122	123	124	125	126	127	128	129	130	131	132	133	134	135	136	137	138	139	140
P					Pos.con.	159	158	157	156	155	154	153	152	151	150	149	148	147	146	145	144	143	142	141

Figure S11. Microfractionation: General μ-fractionation plate layout. Column 1 contains cation adjusted Mueller Hinton II Medium without test strain as contamination control; Columns 2+3 contain antibiotic standard; Column 4 contains cells/spores in medium without antibiotic or extract fractions as growth control; Area AH05- AH24: Collected fractions from the first injection replicate; Area IP05- IP24: Collected fractions from the second injection replicate. Numbers indicate fraction count for the two injection replicates.

Reference

1. Hoshino, T. Description of Two New Species in the Genus *Agelas* (Demospongia) from Zamami Island, the Ryukyus, Japan. *Proc. Jap. Soc. Syst. Zool.* **1985**, *30*, 1–10.
